# Effects of Process Parameters on Pulsed Laser Micromachining for Glass-Based Microfluidic Devices

**DOI:** 10.3390/ma18112657

**Published:** 2025-06-05

**Authors:** Mrwan Alayed, Nojoud Al Fayez, Salman Alfihed, Naif Alshamrani, Fahad Alghannam

**Affiliations:** 1 Microelectronics and Semiconductors Institute, King Abdulaziz City for Science and Technology (KACST), Riyadh 12354, Saudi Arabia; malayed@kacst.edu.sa (M.A.); salfihed@kacst.edu.sa (S.A.); 2 Advanced Diagnostics and Therapeutics Institute, Health Sector, King Abdulaziz City for Science and Technology (KACST), Riyadh 12354, Saudi Arabia; nalfayez@kacst.edu.sa

**Keywords:** microfluidics, glass micromachining, microchannel, pulsed laser, pulse duration, process parameters, pulsed laser micromachining

## Abstract

Glass-based microfluidic devices are essential for applications such as diagnostics and drug discovery, which utilize their optical clarity and chemical stability. This review systematically analyzes pulsed laser micromachining as a transformative technique for fabricating glass-based microfluidic devices, addressing the limitations of conventional methods. By examining three pulse regimes—long (≥nanosecond), short (picosecond), and ultrashort (femtosecond)—this study evaluates how laser parameters (fluence, scanning speed, pulse duration, repetition rate, wavelength) and glass properties influence ablation efficiency and quality. A higher fluence improves the material ablation efficiency across all the regimes but poses risks of thermal damage or plasma shielding in ultrashort pulses. Optimizing the scanning speed balances the depth and the surface quality, with slower speeds enhancing the channel depth but requiring heat accumulation mitigation. Shorter pulses (femtosecond regime) achieve greater precision (feature resolution) and minimal heat-affected zones through nonlinear absorption, while long pulses enable rapid deep-channel fabrication but with increased thermal stress. Elevating the repetition rate improves the material ablation rates but reduces the surface quality. The influence of wavelength on efficiency and quality varies across the three pulse regimes. Material selection is critical to outcomes and potential applications: fused silica demonstrates a superior surface quality due to low thermal expansion, while soda–lime glass provides cost-effective prototyping. The review emphasizes the advantages of laser micromachining and the benefits of a wide range of applications. Future directions should focus on optimizing the process parameters to improve the efficiency and quality of the produced devices at a lower cost to expand their uses in biomedical, environmental, and quantum applications.

## 1. Introduction

Microfluidics is a technology that manipulates or processes small volumes of fluids, typically ranging from 10^−6^ to 10^−18^ L, within microscale structures [1]. This technology allows for the miniaturization of entire laboratory systems onto chips that are only a few centimeters wide, enabling the detailed observation and analysis of various properties, including physical, chemical, optical, and biological properties [2]. Microfluidic devices offer several benefits: they require less fluid, reduce analysis time, lower costs, and increase safety compared to traditional methods [3]. These advantages make microfluidics critical for a wide range of applications, such as diagnostics, environmental monitoring, and drug discovery [3].

The development of microfluidics has been influenced by progress in materials and fabrication methods over many years [4]. Initially, microfluidic devices were primarily made from inorganic substances like glass and silicon, utilizing photolithography and wet/dry etching techniques adapted from microelectromechanical system processing [5]. Glass, such as fused silica, borosilicate, and soda–lime, is valued for its outstanding optical clarity, chemical inertness, thermal stability, and mechanical durability [6]. Although silicon provides excellent precision in microfabrication and is compatible with MEMS and microfluidics technologies, it is not ideal for optical detection due to its opacity in the visible spectrum [7]. As the field has progressed, organic materials, like polydimethylsiloxane (PDMS) and polymethylmethacrylate (PMMA), have become more favored due to their straightforward fabrication methods (e.g., soft lithography), flexibility, and cost-effectiveness, as detailed in our earlier research [8,9]. Nonetheless, glass-based microfluidic devices continue to be an attractive option, particularly for biomedical applications, such as point-of-care diagnostics and cell analysis [10,11].

Traditional methods for fabricating glass-based microfluidic devices utilize photolithography to create microchannel patterns on photoresist-coated glass substrates, hydrofluoric acid for wet chemical etching to form these channels, and soft lithography for molding and bonding PDMS structures to glass [5,12,13]. Although these techniques yield precise geometries, enable replication, and ensure material compatibility, they come with certain drawbacks. Their complexity, high production costs, and the requirement for specialized cleanroom environments and hazardous chemicals are significant limiting factors [11,14]. Additionally, these methods often involve multiple intricate procedures, such as precise alignment, stacking, and bonding, which elevate the risk of defects and delays in production while also constraining design flexibility and the capacity to construct complex structures [2].

Recent methods for glass-based microfluidic fabrication have improved accessibility and efficiency. For example, emerging 3D printing approaches using glass-compatible materials or sacrificial molds enable complex structures, though further improvements in resolution are needed [6,15]. Moreover, the development of laser techniques has greatly advanced laser-based fabrication, such as precise bio-template creation, neural microstructure fabrication, and advanced material processing [16,17,18]. Laser micromachining is utilized in glass-based microfluidic fabrication because it enables higher precision, finer feature resolution, better depth control, minimal thermal damage, and increased flexibility in processing a wide range of materials. This makes it possible to rapidly and reproducibly fabricate complex microstructures for applications in fields such as medical devices, electronics, and microfluidics [19,20].

Laser micromachining is a manufacturing technique that employs laser beams to remove small amounts of material, enabling the creation of intricate structures for applications in microfluidics, medical devices, and electronics [21]. There are two main types: direct micromachining, where the laser cuts materials like glass for robust applications, and indirect micromachining, where energy is transmitted through a different medium, making it suitable for more delicate tasks such as bioprinting [22,23]. This level of precision and adaptability positions laser micromachining as a superior choice over traditional methods, particularly for glass-based microfluidic systems that require high accuracy [24]. Laser micromachining is governed by process parameters such as laser fluence, scanning speed, pulse duration, and wavelength, allowing for high ablation efficiency and quality [25]. The mechanisms of laser ablation are largely determined by the pulse duration. Ultrashort pulses employ non-thermal, “cool” methods that minimize heat-related damage. Short pulses are sufficiently brief to restrict heat diffusion into the surrounding material. In contrast, longer pulses rely on thermal effects, which can cause undesirable side effects such as cracking and melting [26].

Though several reviews have been published on laser micromachining, microfluidic materials, fabrication, and applications, a significant gap exists in comprehensive reviews specifically analyzing the impacts of process parameters on the efficiency and quality of laser micromachining of glass. Thus, this review explores the effects of process parameters on the ablation efficiency and quality of glass microstructures. Due to the different mechanisms of laser ablation between nanosecond and femtosecond lasers, we categorize the pulsed laser micromachine systems into three pulsed laser regimes (long, short, and ultrashort). Within each category, we surveyed the studies and analyzed various processing parameters, offering insights into optimizing laser processes for future glass-based microfluidic devices. We discuss the impact of laser process parameters, focusing on fluence, scanning speed, pulse duration, repetition rate, and wavelength, alongside the type of glass, on the quality and efficiency of glass micromachining.

This review is composed of the following sections. Section 2 discusses microfluidic materials with a focus on glass’ benefits, compares fabrication methods, classifies laser techniques into direct and indirect types, and describes the review’s approach to evaluating pulsed laser parameters. Section 3 categorizes the reviewed laser ablation techniques into three groups (long, short, and ultrashort) based on pulse duration. For each category, we examine the influence of laser fabrication parameters on the results of glass microchannels and microcavities, along with process parameter optimization. Section 4 lists the benefits (e.g., high precision, minimal thermal damage, rapid prototyping) and highlights the applications of laser-based glass microfluidics. Lastly, Section 5 presents the conclusion and provides recommendations for future directions.

## 2. Background and Methodology

Microfluidic devices can be fabricated from various materials, each providing unique optical, electrical, chemical, and mechanical properties suited for specific applications [2,27]. Section 2.1 summarizes the features of the main materials, particularly glass. Section 2.2 briefly discusses the main fabrication methods of glass-based microfluidic devices and compares their characteristics. In addition, Section 2.3 focuses on the types of laser techniques used to fabricate glass-based microfluidic devices. Shifting focus, Section 2.4 outlines the review’s methodology, detailing the systematic literature search on pulsed laser regimes and the selected process parameters, like fluence and scanning speed, which ensured a structured analysis.

### 2.1. Materials of Microfluidic Devices

Materials for microfluidic devices are generally classified into four main categories: inorganic materials (e.g., glass, silicon, ceramics), polymer-based materials (e.g., PDMS, polymethylmethacrylate (PMMA)), hybrid materials (e.g., glass–polymer combinations), and composite materials (e.g., Flexdym polymer with polycarbonate membranes) [12,27,28]. Early microfluidic devices were developed on inorganic substrates, with types of glass, like fused silica and Foturan, providing specific advantages over materials like silicon, particularly in situations requiring precise optical measurements [29]. However, the material selection for microfluidic system fabrication depends on the manufacturing technique and the specific application requirements [12,30].

In recent years, interest in inorganic materials, especially glass, has surged due to their favorable attributes, which are ideal for precise applications [11]. Therefore, optical transparency, thermal stability, mechanical stability, chemical inertness, and biocompatibility are all distinctive features that make glass a leading choice for microfluidic substrates across various applications, as follows [6,11]. First, glass’s exceptional optical transparency, with up to 90% transmission (e.g., fused silica), enables optical detection over a broad spectrum from UV to near-IR, with low background fluorescence and minimal optical nonlinearity or dispersion [5,11]. Second, glass’s thermal stability and high temperature resistance are important for high-temperature processes, like the polymerase chain reaction (PCR) in DNA amplification [11]. Third, glass’s mechanical stability provides durability under high pressure and also allows for the creation of smooth surfaces and very small channels (down to 10 nm), which is critical for single-cell manipulation and high-resolution imaging in optofluidic sensors [6]. Fourth, chemical inertness ensures biocompatibility and minimal contamination, outperforming PDMS in biomedical uses like drug delivery, where any interaction with the substrate could alter results [11]. Overall, glass has been favored as a substrate for microfluidic devices due to its excellent optical, thermal, mechanical, and chemical properties [11].

Several types of glass have been used as substrates in microfluidic devices due to their unique properties [5]. Firstly, fused silica is praised for its exceptional optical properties, including a broad transmission range and low autofluorescence, making it ideal for optofluidic applications [31]. Secondly, borosilicate glass is valued for its thermal and chemical resistance, which makes it ideal for microreactors and micromixers [5]. For instance, Pyrex (Corning 7740) is noted for its thermal shock resistance, which is ideal for bio-applications that require robustness and high resistance to temperature fluctuation, such as PCR and lab-on-chip devices [5,32]. Thirdly, although less resistant than others, soda–lime glass is commonly used due to its affordability in the form of disposable microfluidic devices for basic mixing and reaction tasks, such as academic demonstrations or preliminary screening [33]. Fourthly, quartz is selected for its superior optical properties, which are crucial in applications demanding high optical clarity, making quartz ideal for optofluidic systems and optical spectroscopy over the range of UV-NIR [23]. Fifthly, ultra-thin glass provides flexibility where thickness is a constraint, so it is proper for wearable sensors and implantable devices [11]. Lastly, Foturan glass, a photo-structurally glass-ceramic, allows for the creation of complex 3D structures through UV light patterning and etching [34,35]. These types of glass offer various options, each designed to meet the diverse needs of microfluidic device design and functionality.

### 2.2. Fabrication Methods of Glass-Based Microfluidic Devices

**Figure 1:**
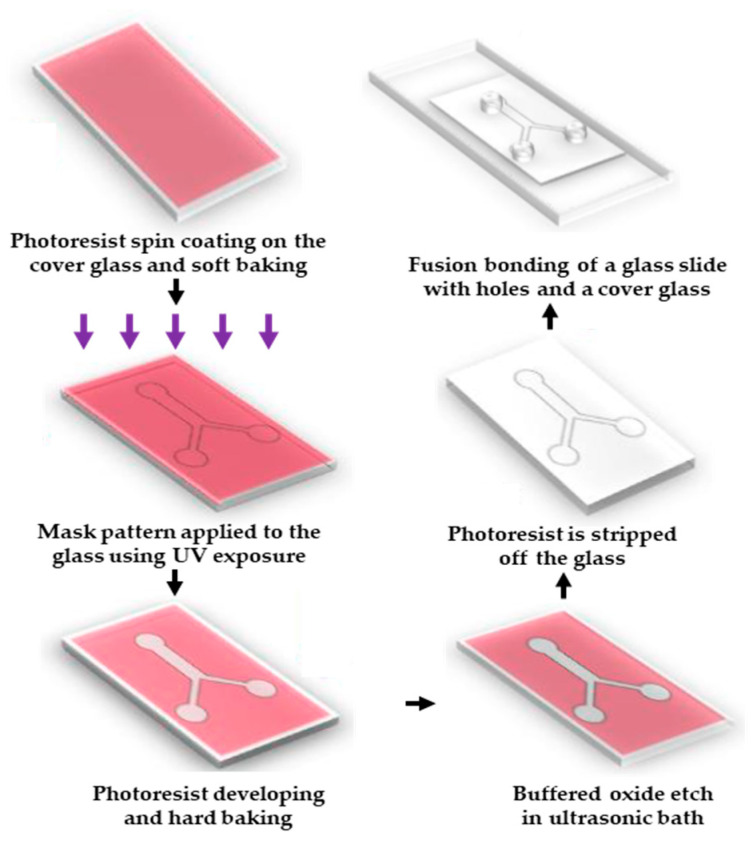
Production of glass microfluidic chips using wet etching and fusion bonding methods.

Despite these advantages, glass presents significant challenges in fabricating microfluidic devices. Traditional manufacturing methods for glass chips are complex, involving intricate processes, like alignment and stacking (Figure 1), which are more demanding than those for materials such as PDMS or PMMA [12,36]. These processes require specialized equipment and multiple manufacturing steps, leading to the high cost and lengthy production times of glass-based microfluidic devices [37]. As a result, producing microfluidic devices with glass substrates can be time-consuming and costly. To overcome these challenges, several strategies have been developed to simplify the fabrication of glass-based microfluidic devices [38].

Several methods for fabricating glass-based microfluidic devices have been demonstrated, including photolithography, wet chemical etching, soft lithography, 3D printing, and laser ablation [17]. These key fabrication techniques for glass-based microfluidic microstructures are summarized in Table 1. First, photolithography creates a pattern on a photoresist layer using UV light through a mask, followed by etching to form microchannels [12]. Second, wet chemical etching is another established technique, where etchants like hydrofluoric acid or potassium hydroxide selectively remove glass [5]. This method is often used alongside photolithography to define microstructures [34]. Third, soft lithography involves creating molds from materials such as PDMS (polydimethylsiloxane) and then using these molds to shape or bond with glass [37]. Fourth, a recent development in microfluidic fabrication is the use of 3D printing technologies, which offer rapid prototyping, complex geometries, and leverage glass’s superior properties [39]. However, 3D printing still needs enhanced scalability and precision for broader adoption to overcome its limitations, such as its lower resolution (~10 µm), surface roughness, and high equipment costs [40]. Additionally, laser ablation has emerged as a promising method for fabricating glass-based microfluidic devices [41]. This technique utilizes lasers, particularly pulsed lasers, to directly remove or modify glass material, facilitating the creation of complex microstructures with high precision [34].

### 2.3. Types of Laser-Based Fabrication Methods for Glass-Based Microfluidics

Laser-based fabrication methods for glass microfluidics can create precise microstructures, such as microchannels in glass substrates. These methods can be categorized into direct and indirect approaches, depending on whether the laser directly removes material or is assisted by additional processes, like chemical etching or plasma-assisted techniques [42,43].

Indirect methods utilize lasers to initiate or enhance a secondary process, typically chemical etching, to form microstructures [23]. Thus, indirect methods place a greater emphasis on the surface smoothness of microfluidic features, rather than on process speed. For example, Laser-Induced Backside Wet Etching (LIBWE) is an indirect method that employs a UV laser (e.g., 355 nm) to heat an absorbing liquid (e.g., toluene), which drives hydrofluoric acid (HF) etching to create smooth 2D channels (20–50 μm) in borosilicate [5]. Additionally, Femtosecond Laser-Induced Chemical Etching (FLICE) is another indirect method that modifies glass with a femtosecond laser, followed by HF etching to create smooth 3D channels (10–200 μm) in fused silica [10,44]. Generally, indirect methods prioritize surface quality; however, they necessitate multi-step processes and the use of hazardous chemicals [11].

On the other hand, direct methods include laser ablation and laser direct writing (LDW) [45]. Laser ablation has three forms: thermal ablation, photochemical ablation, and multiphoton ablation. Thermal ablation uses CO_2_ lasers (10.6 μm) to vaporize glass (e.g., borosilicate), forming channels (>100 μm) quickly but with rough surfaces [46]. Photochemical ablation employs UV lasers, such as excimer lasers (e.g., 193 nm), to etch fine 2D features (1–5 μm) in glass (e.g., fused silica) with minimal heat [47]. Multiphoton ablation via femtosecond lasers (~800 nm) enables 3D structuring (e.g., buried channels) with sub-micrometer precision and minor thermal damage [48]. LDW uses femtosecond lasers, crafts intricate 3D patterns in fused silica, and offers high-resolution, but slower, processing [34]. Direct methods, like femtosecond ablation and LDW, generally lead to speed and versatility for complex designs, while indirect methods, like FLICE and LIBWE, offer smooth finishes.

The choice between the two techniques for laser micromachining depends on whether speed and complexity (direct) or surface quality (indirect) are prioritized. This review emphasizes direct laser ablation because of its growing adoption in industrial and academic settings, driven by advancements in ultrafast laser technology. This review concentrates on direct methods, offering a framework and guidance for utilizing pulsed lasers to tackle challenges in microfluidic fabrication, including throughput optimization and surface quality improvement. It examines the direct laser ablation technique, emphasizing the application of pulsed lasers across different processing parameters.

### 2.4. Methodology

This systematic review comprehensively examines the effects of process parameters on pulsed laser micromachining for glass-based microfluidic devices. The review systematically analyzes and synthesizes existing research in the field, categorizing findings based on different laser pulse regimes and specific processing parameters. A comprehensive literature search was conducted on the Web of Science and Google Scholar, focusing primarily on peer-reviewed research articles (2018–2024) and using keywords such as “pulsed laser”, “laser ablation”, “glass microfluidics”, and “glass micromachining”. Our search targeted experimental studies quantifying ablation efficiency (e.g., depth, removal rate) or quality metrics (e.g., surface roughness, heat-affected zones) for glass substrates, with pulse durations spanning from 1 fs to 1 ms. The eligible studies examined the effects of process parameters on the quality and efficiency of pulsed laser micromachining for glass-based microfluidic devices. The exclusion criteria removed theoretical models, non-English publications, and studies lacking empirical validation. The final search was conducted in April 2025 and also included some online reports, electronic books, conference proceedings, dissertations, and theses.

The review systematically analyzed and synthesized existing research in the field, categorizing findings based on different laser pulse regimes (long (≥nanosecond), short (picosecond), and ultrashort (femtosecond)) and specific processing parameters (fluence, scanning speed, pulse duration, repetition rate, wavelength, and glass type). This structured approach ensures transparency, enabling the direct comparison of nanosecond, picosecond, and femtosecond regimes. The review employs a comparative approach, using detailed tables to clearly present findings from multiple studies and provide a comprehensive synthesis of current research. It evaluates both the quality and efficiency of laser micromachining processes and systematically builds understanding through a step-by-step analysis in each section.

## 3. Direct Pulsed Laser Processing for Glass Microfabrication

**Figure 2:**
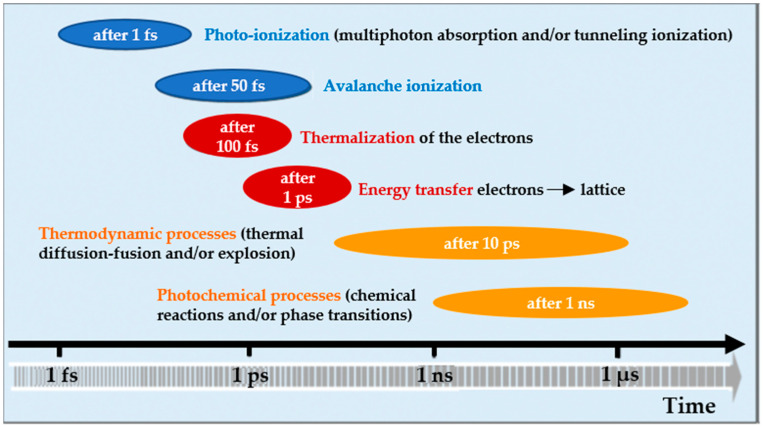
The occurrence of various physical phenomena that govern the material removal process across different timescales during laser-material interaction [50]. Adapted with permission from reference [50].

Laser systems generate beams through the stimulated emission of radiation [49]. The three essential components for laser operation are the gain medium, laser cavity, and pump source. Lasers can operate in a continuous wave (CW) or a pulsed mode, with the pulsed mode further categorized into three regimes: long-pulse (≥nanoseconds), short-pulse (picoseconds), and ultrashort-pulse (femtoseconds) [50,51,52,53]. Lasers’ pulse durations are critical for fabricating microfluidic components, such as microchannels and cavities [29]. The pulse duration primarily influences the material removal mechanism, as illustrated in Figure 2. Microchannels are key components that define microfluidic devices, with glass frequently being chosen as the preferred substrate material [5]. Consequently, enhancing the efficiency of microchannel fabrication on glass is essential. Recent studies have focused on using pulsed lasers for microchannel fabrication, as they offer high ablation efficiency and quality with minimal thermal impact [54,55,56].

Ablation efficiency and quality are important for achieving high-performing, reliable, and cost-efficient glass-based microfluidic devices [57]. Ablation efficiency refers to how effectively material is removed during the ablation process [50]. As demonstrated in earlier research, ablation efficiency is often defined by the specific removal rate, which can be determined by measuring the depth of ablated microchannels [58]. Efficiency measures how much material is removed per unit of energy or laser fluence. On the other hand, ablation quality assesses the overall condition and characteristics of the surface after the process, including surface smoothness, the precision of the etching features (e.g., microchannels), and the absence of defects like cracks, roughness, or thermal damage [29]. A high ablation quality ensures that the material removal is accurate and clean, with minimal thermal impact on the surrounding material, which is crucial for applications requiring high precision, such as microfluidic device fabrication [7]. Both the efficiency and quality of ablation are influenced by factors such as fluence, scanning speed, pulse duration, repetition rate, wavelength, and material types [33,59]. Fluence, representing the energy delivered per unit area, directly impacts material removal [60]. The scanning speed affects the interaction time between the laser and the material, which influences etching depth [61]. The pulse duration impacts thermal effects and energy deposition rates [62]. The repetition rate affects heat accumulation and ablation efficiency [63]. The laser wavelength plays a role in the absorption characteristics of the material [64]. Different types of glass respond differently to laser irradiation, further impacting process efficiency [65]. Overall, the abovementioned factors usually have trade-off effects on ablation efficiency and quality [65].

Achieving an optimal balance between ablation efficiency and etching quality is crucial in glass microfluidic device fabrication, as it directly impacts both the scalability and performance of advanced lab-on-chip systems. Enhanced ablation efficiency enables faster production and lowers manufacturing costs, making large-scale device fabrication more practical. At the same time, a high etching quality is essential for generating smooth, defect-free microchannels that support precise fluid manipulation and maintain the optical clarity necessary for sensitive medical diagnostics and chemical analyses [66]. Therefore, understanding and optimizing ablation efficiency and quality are essential for successfully producing microfluidic devices with the desired characteristics and performance. This section discusses the efficiency and quality of glass microfabrication across three pulse duration regimes: long-pulse, short-pulse, and ultrashort-pulse lasers, considering the various factors outlined in Table 2.

### 3.1. Long-Pulse Laser Micromachining of Glass

**Figure 3:**
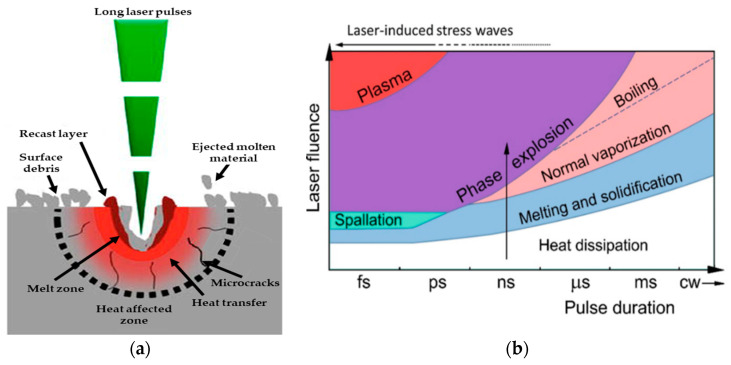
(**a**) Long-pulse laser ablation interaction with a target material [75]; (**b**) Laser-induced thermal effects: heat transfer, stress generation, melting and solidification, normal vaporization, and phase transition, influenced by pulse duration and laser fluence [74]. Adapted with permission from references [74,75].

In long-pulse laser ablation, material removal primarily occurs through thermal processes [67,68]. When ≥nanosecond pulses are applied to a target, such as glass, photons excite electrons and induce molecular vibration–rotation, transferring energy to the local lattice as thermal energy, known as thermal diffusion, which primarily depends on the thermal diffusivity (*D*) of the material [69]. For instance, fused silica, composed almost entirely of silicon dioxide (SiO_2_), exhibits a higher thermal diffusivity (approximately 0.75–0.85 mm^2^/s) compared to borosilicate glass (about 0.64 mm^2^/s) [70]. As a result, fused silica dissipates heat more rapidly, which helps minimize the duration of localized heating and reduces the risk of thermal stress and crack formation during rapid heating and cooling cycles. This property makes fused silica especially suitable for high-precision microfluidic applications, despite its higher cost relative to other glass types [71]. The thermal diffusion process heats the material to its melting point and raises its temperature to a level where the atoms acquire enough energy to transition into a gaseous state [72]. There is sufficient time for a thermal wave to spread through the glass [73]. The molten material is partially expelled from the cavity by vapor and plasma pressure, forming a microchannel or microcavity [74]. However, some material remains near the surface, forming a recast layer, as shown in Figure 3a [74,75].

Numerous literature reviews have explored the fundamental mechanisms of long-pulse laser interactions with matter and the micromachining processes [76,77]. These processes can be summarized in four main steps—heat dissipation, melting and solidification, normal vaporization, and boiling—which depend on pulse duration and laser fluence, as shown in Figure 3b [74]. Therefore, when using long-pulse laser micromachining, it is essential to balance high ablation efficiency with preserving surface integrity by evaluating the effects of laser process parameters.

#### 3.1.1. Effects of Laser Fluence in Long-Pulse Micromachining

The impact of laser fluence on ablation efficiency is a key factor in laser micromachining, as variations in fluence strongly influence glass removal rates and etching depths. Several studies have investigated the relationship between process efficiency and fluence using micro- and nanosecond-pulse lasers. A study examined the ablation efficiency as a function of fluence in quartz glass using CO_2_ laser pulses (*t_p_ =* 300 ns, *λ* = 10.6 μm, *PRR* = 20 kHz, *d_L_* = 240 μm, and *V_scan_* = 20 mm/s), with fluence ranging from 1.12 to 7.18 J/cm^2^ [78]. The experiments demonstrated an increase in removal effectiveness with higher fluence, with the efficiency reaching 18 × 10^−3^ mm^3^/J at 7.18 J/cm^2^, compared to only 0.7 × 10^−3^ mm^3^/J at a fluence of 1.12 J/cm^2^. The same approach was reported for diamond samples ablated with a range of fluences from 11.41 to 47.91 J/cm^2^ using a nanosecond laser (*t_p_ =* 12 ns, *λ* = 355 nm, *PRR* = 30 kHz, and *V_scan_* = 5 mm/s) [79]. When the laser fluence is below 28.29 J/cm^2^, the material removal rate of the diamond increases gradually (from 0.45 × 10^−10^ to 0.75 × 10^−10^ g/pulse) due to gentle ablation. However, once the laser fluence exceeds 28.29 J/cm^2^, the material removal rate increases rapidly, reaching 3.34 × 10^−10^ g/pulse at 47.91 J/cm^2^. The increase is due to the greater energy delivered at a higher fluence, which leads to higher local heat accumulation, reducing the threshold fluence and the optical penetration depth [78]. The removal depth of fused silica was also investigated as a function of fluence in the range of 3 to 5.8 J/cm^2^ using a CO_2_ laser (*t_p_ =* 45 μs, *λ* = 10.6 μm, *PRR* = 1 kHz, *d_L_* = 300 μm, and *V_scan_* = 20 mm/s) [80]. The results showed an approximately exponential increase in the etching depth as the fluence increased, with the depth reaching up to 10 μm at 5.8 J/cm^2^, compared to only 0.04 μm at 3 J/cm^2^. Another study explored the removal depth of soda–lime glass at high fluence levels (20 to 132.3 J/cm^2^) using an Nd:YAG laser (*t_p_ =* 30 ns, *λ* = 355 nm, *PRR* = 5 kHz, *d_L_* = 15 μm, and *V_scan_* = 20 mm/s) [81]. The findings revealed that the ablation depth increased from 12 μm to 50 μm as the laser fluence rose from 20 to 100 J/cm^2^, after which the depth began to saturate due to the accumulation of ablated debris, limiting further laser interaction with the glass substrate. The limitation of depth saturation should be considered when fabricating high-depth microchannels for fluidic devices using high fluence. However, this limitation can be avoided using one of several techniques, such as multi-pass ablation, vacuum suction, ultrasonic cleaning, and high-pressure gas jets [66,82]. Overall, the results indicate that laser fluence influences ablation efficiency and etching depth, where higher fluences enhance material removal rates but may lead to saturation and debris accumulation.

**Figure 4:**
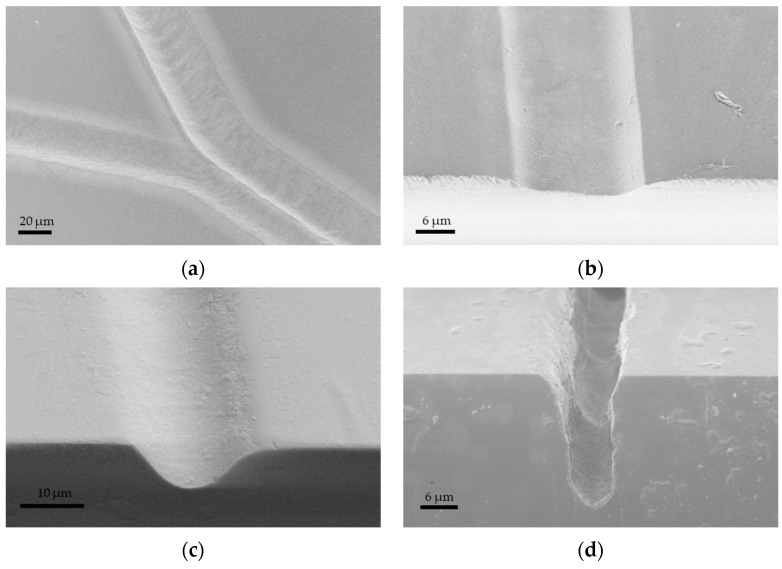
(**a**) An SEM image of two adjacent channels merging into an asymmetric trench in a Borofloat glass substrate. The stage’s speed fluctuation causes a ripple at the bottom of the channel. (**b**) Smooth ablation is achieved by optimizing the motion parameter. (**c**) An SEM image of a 6 μm wide trench. (**d**) An SEM image of a deeper trench fabricated by increasing the laser pulse energy or the number of repeated ablations. The channels in these images were manufactured using a laser pulse energy of 78 μJ with an Nd:YAG laser (*t_p_* = 10 ns, *λ* = 266 μm, *PRR* = 4.5 kHz, *d_L_* = 6 μm, and *V_scan_* = 0.2 mm/s for (**a**,**c**) and 0.6 mm/s for (**b**,**d**) [84]. Adapted with permission from reference [84].

In addition to the apparent influence of high pulse fluence on removal depth, high fluence can also affect ablation quality, potentially causing thermal damage, rough surface finish, microcracks, and debris formation. Temmler et al. used a CO_2_ laser (*t_p_* = 300 ns, *λ* = 10.6 μm, *PRR* = 150 kHz, *d_L_* = 240 μm, and *V_scan_* = 750 mm/s) to explore how fluence affects the etching quality of quartz glass [78]. It was found that the surface roughness (Sa) increased to 0.75 μm as the laser fluence rose from 0.80 to 2 J/cm^2^ due to pulse stability issues and heat accumulation. Furthermore, Wang and Zheng reported that clear surface melting and solidification cracks form surrounding microchannels on a soda–lime substrate when the energy deposition rate exceeds 6.0 J/(cm^2^·s), whereas crack-free channels form at a lower energy using a CO_2_ laser (*λ* = 10.6 μm, *PRR* = 5 kHz, *d_L_* = 347 μm, and *V_scan_* = 2000 mm/s) [83]. A similar approach was observed using low laser pulse energy (78 μJ) with an Nd:YAG laser (*t_p_* = 10 ns, *λ* = 266 nm, *PRR* = 4.5 kHz, *d_L_* = 6 μm, and *V_scan_* = 0.2 and 0.6 mm/s) to fabricate Borofloat microfluidic channels [84]. The results showed high-quality, crack-free microchannels (Figure 4a), with periodic ripple-like fluctuations at the trench bottom attributed to stage speed fluctuations. By optimizing the stage settings, a smoother surface was achieved (Figure 4b,c). However, when the laser pulse energy was increased, deeper trenches were created (a higher aspect ratio), but the surface became rough, and debris appeared near the sidewalls (Figure 4d), likely due to the redeposition of ablated glass. While an increase in fluence enhances efficiency, the findings suggest that a lower fluence is more desirable for attaining high-quality fabrication with minimal side effects. However, it is important to note that this may result in a reduction in process efficiency. Therefore, a trade-off between depth and quality should be considered when fabricating microfluidic channels with varying laser fluence. If increased removal effectiveness is required, auxiliary methods, such as air or water flow, can be employed during the ablation process to minimize the heat accumulation effect and ablation debris in the targeted area, as discussed in a recent review study [85].

#### 3.1.2. Effects of Scanning Speed in Long-Pulse Micromachining

The impact of scanning speed on the processing properties was assessed by comparing the experimental results obtained with different scanning speeds. Chen and Darling studied the effect of scanning speed (5–10 mm/s) on the removal rate of Pyrex using a nanosecond-pulse Nd:YAG laser (*λ* = 266 nm, *PRR* = 5 kHz, and *F* = 24 J/cm^2^) [86]. The experiments showed an increase in the removal rate from 10 to 18 μm/pulse as the scanning speed increased from 5 to 10 mm/s. This increase may result from a reduction in negative overheating effects, which can lead to material melting. Another study on K-PSFn214 glass machining using a CO_2_ laser (*t_p_* = 45 μs, *λ* = 10.6 μm, *PRR* = 20 kHz, *d_L_* = 400 μm, and *V_scan_* = 100 to 600 mm/s) investigated the impact of scanning speed on ablation depth [22]. It found that as the scanning speed increased, the microchannel depth decreased, with the maximum depth (approximately 10 μm) achieved at 100 mm/s, compared to less than 4 μm at 600 mm/s. In a similar study, the effect of scanning speed on etching depth in microscope glass substrates was examined using a CO_2_ laser system (*P_p_* = 4.5 W) [21]. The findings revealed that as the scanning speed increased, the microchannel depth decreased, reaching a minimum of 22.4 μm at 25 mm/s, compared to 55.8 μm at lower speeds (5 mm/s). This can be explained by the fact that the scanning speed directly affects the exposure time of the laser beam on the substrate [25]. Slower speeds result in more laser energy being absorbed, increasing the heat applied to the substrate and, consequently, the depth of the cut. Overall, slower scanning speeds increase ablation depths and removal rates by allowing more-prolonged laser exposure, but this can cause overheating and material melting, which affect ablation quality, as will be discussed next.

**Figure 5:**
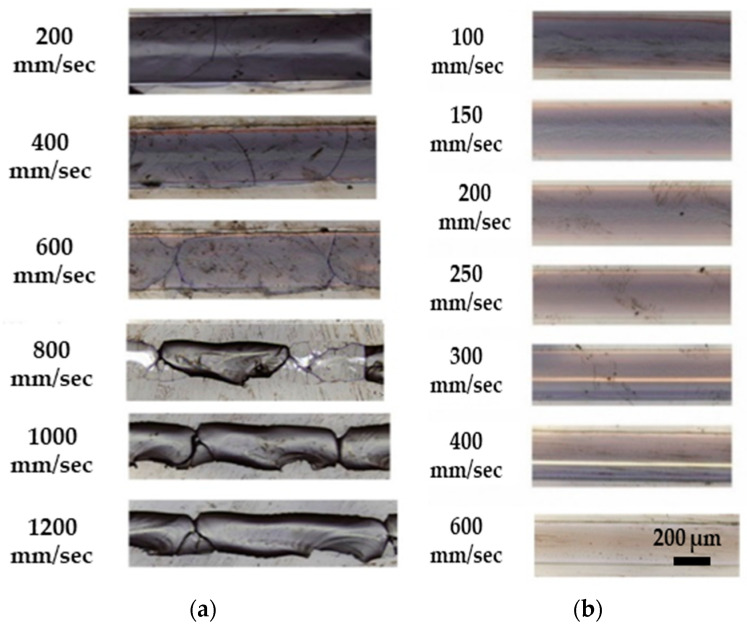
Top view images of (**a**) direct laser microchannels without the use of glass preheating, displaying noticeable cracks and thermal damage; (**b**) high-quality microchannels achieved with glass preheating at 250 °C after varying CO_2_ laser scanning speeds, utilizing a power of 30 W, a frequency of 20 kHz, and a beam diameter of 400 μm [22]. Adapted with permission from reference [22].

Several studies have explored the impact of scanning speed on the quality of microchannel structures. For instance, Yasman and co-workers utilized a CO_2_ laser system (*t_p_* = 50 μs, *λ* = 10.6 μm, *PRR* = 20 kHz, *d_L_* = 400 μm, *V_scan_* = 100 and 1200 mm/s, and *P_p_ =* 30 W) to investigate the effect of scanning speed on etching quality [22]. The study reported visible microcracks in the microfluidic channels formed in the K-PSFn214 substrate under all the applied scanning speeds (Figure 5a). This phenomenon can be attributed to thermal shockwaves caused by intense laser irradiation. Cracks may also form when thermal stress surpasses the glass critical threshold. Exposure to high-intensity laser irradiation causes localized heating, creating significant temperature gradients within the material. These gradients lead to differential expansion and contraction, and if the thermal stress exceeds the glass critical limit, cracks can initiate and spread along the microchannels [87]. To maintain ablation efficiency and avoid poor quality, the study suggested preheating the glass to 250 °C before laser processing. The preheating significantly improved the quality of the microchannels, as shown in Figure 5b, by preventing sudden temperature spikes and ensuring the precise control of the glass surface temperature. The same side effects from the removal process with a CO_2_ laser on thin glass were reported in the Brusberg et al. study [88]. The thermal effect of the laser on the glass induces mechanical stress in the ablation area, leading to the formation of cracks. To mitigate the thermal effects, local preheating of the removal area was applied, with only 13% of the preheated area showing cracks. Additionally, Bondarenko and co-authors investigated the roughness of microchannels and reservoirs fabricated in fused silica using a CO_2_ laser (*λ* = 10.6 μm, *PRR* = 8 kHz, *d_L_* = 62 μm, *V_scan_* = 50–65 mm/s, and *P_p_ =* 5.8 W) [89]. The findings indicated that a greater pulse overlap (90%) (lower scanning speed) increased the surface roughness from 350 nm (at 80%) to 1500 nm. The reported studies indicate that reducing the scanning speed increases the removal depth; however, this enhancement comes with a reduction in etching precision. Therefore, a trade-off between depth and quality should be considered when fabricating microfluidic channels with a high aspect ratio.

#### 3.1.3. Effects of Pulse Duration in Long-Pulse Micromachining

Extended pulse durations, in the micro- and nanosecond range, significantly influence the ablation depth in glass micromachining [90]. A recent study examined the impact of extended microsecond pulse durations on the removal depth of fused silica using a CO_2_ laser (*t_p_* = 54–185 μs, *λ* = 10.6 μm, *PRR* = 1 kHz, *d_L_* = 300 μm, and *P_p_* = 25 W) [80]. The results showed that as the pulse duration increased from 54 to 185 μs, the etching depth rose from approximately 3.5 nm to over 8.2 μm. The effect of a wider pulse duration range (300–1000 μs) on the ablation depth of fused silica was investigated using a CO_2_ laser (*λ* = 10.6 μm, *PRR* = 130 kHz, *d_L_* = 266 μm, and *P_p_* = 85 W) [91]. The measurements indicate an increase in crater depth as pulse duration increases, where a depth of 44 μm was recorded at 1000 μs pulses compared to only 5 μm when 300 μs pulses were applied. This can be attributed to the fact that an extended pulse duration (in the range of μs) increases the amount of thermal energy imparted to the material. The increased heating time can result in more significant thermal damage to the glass, allowing it to melt, ablate, or vaporize over a larger area, which increases the ablation depth [92]. Based on these findings, deep channels for microfluidic devices can be fabricated by extending microsecond laser pulses from tens to hundreds of microseconds while considering the thermal effects that may arise and impact removal quality.

The extended exposure time (longer pulses) may lead to a rougher surface finish than shorter pulse durations. The effect of pulse duration (ranging from 1 μs to 200 μs) on the removal quality of fused silica and borosilicate glass was studied using a CO_2_ laser (*λ* = 10.6 μm, *PRR* = 200 Hz, *d_L_* = 35 μm, and *P_p_* = 30 W) [93]. It was observed that shorter laser pulses produce a smaller heat-affected zone (HAZ) within the irradiated area, reducing the potential for cracking in the glass substrate. This is particularly important for borosilicate glass, which has a higher coefficient of thermal expansion (3.3 × 10^−6^ K^−1^) compared to fused silica (0.5 × 10^−6^ K^−1^) [39,94]. To minimize the risk of cracking in borosilicate glass, it is suggested to use a pulse length of ≤10 μs. The same approach was observed by Zhang et al., who used CO_2_ laser pulses with pulse lengths from 5 μs to 90 μs (*λ* = 10.6 μm, *PRR* = 500 Hz, *d_L_* = 90 μm, *V_scan_* = 75 mm/s, and *P_p_* = 100 W) on a fused silica substrate [95]. The evolution of the HAZ shows that its thickness increases from 3 μm to 9.6 μm as the pulse duration extends from 5 μs to 90 μs. The thickness of the HAZ increases significantly with an increase in pulse length. Conical-shaped damage also increases with longer pulse durations. A lower raised rim of only 10 nm is observed for the shorter pulse duration of 6 μs, while the raised rim grows to about 200 nm as the pulse length is increased to 90 μs. Therefore, shortening the pulse length can generate higher-quality microchannels but with less depth, which may result in longer fabrication times for microfluidic channels with a high aspect ratio.

#### 3.1.4. Effects of Repetition Rate in Long-Pulse Micromachining

The laser repetition rate has a significant influence on the ablation process, as higher repetition rates can enhance the material ablation efficiency. Temmler and his co-authors observed a strong impact of frequency on quartz glass micromachining using CO_2_ laser pulses with a 300 ns pulse duration (*λ* = 10.6 μm, *d_L_* = 240 μm, *V_scan_* = 20 mm/s, and *F* = 1.82 J/cm^2^), where repetition rates ranged from 20 kHz to 150 kHz [78]. The results demonstrated that the ablation efficiency increased from 5 × 10^−3^ to 12 × 10^−3^ mm^3^/J as the repetition rate was raised from 20 to 150 kHz. This phenomenon may stem from local heat accumulation at higher pulse repetition rates, leading to a decrease in the threshold fluence, dropping from 0.93 J/cm^2^ at 20 kHz to 0.37 J/cm^2^ at 150 kHz. A recent study also investigated the effect of repetition rates in the range of 200 Hz to 3200 Hz on etching depth using a CO_2_ laser (*t_p_* = 100 μs, *λ* = 10.6 μm, *d_L_* = 300 μm, *V_scan_* = 20 mm/s, and *F* = 3.54 J/cm^2^) and fused silica as the substrate [80]. The study found that increasing the repetition rate from 300 to 3200 Hz resulted in a 500% increase in the microchannel depth. This significant increase was attributed to enhanced local heat accumulation at 3200 Hz, where the material has insufficient time to cool between pulses. Consequently, the thermal buildup accelerates the material’s ability to reach its ablation threshold, enabling deeper material removal [78]. Thus, higher laser repetition rates increase ablation efficiency and microchannel depth in quartz glass and fused silica due to heat accumulation.

The impact of long laser pulses on ablation quality is significantly affected by the repetition rate of the laser because higher repetition rates cause increased thermal accumulation, which can adversely impact precision and surface quality. A study by Varela et al. found that the number of cracks and surface irregularities on soda–lime channels rose sharply for repetition rates above 10 kHz when utilizing an Nd:YVO_4_ laser (*t_p_* = 20 ns, *λ* = 1064 nm, *d_L_* = 20 μm, *V_scan_* = 200 mm/s, and *F* = 78.3 J/cm^2^) [23]. Moreover, the roughness at the bottom of the channels escalated with higher repetition rates, reaching 19 μm at 16 kHz compared to 12 μm at 8 kHz. Therefore, smooth surfaces and walls of microchannels can be achieved using a low repetition rate, avoiding excessive thermal effects.

#### 3.1.5. Effects of Laser Wavelength in Long-Pulse Micromachining

Wavelength is a crucial parameter affecting the optical penetration depth into the sample and the photon energy required for bond breaking. Different wavelengths interact with glass in unique ways due to variations in absorption. Shorter wavelengths, especially in the UV range, are generally absorbed more efficiently by glass, leading to a higher ablation efficiency. Chen and Darling investigated the influence of wavelength on the ablation rates of Pyrex glass and sapphire using near- and mid-UV nanosecond-pulse Nd:YAG lasers (*λ* = 355 and 266 nm, *V_scan_* = 10 mm/s, and *F* = 30 J/cm^2^) [86]. The results demonstrated that etching at 266 nm was primarily driven by a photochemical mechanism, resulting in higher removal rates of 23 and 0.8 μm/pulse for Pyrex glass and sapphire, respectively. In contrast, ablation at 355 nm was achieved through a photothermal process, with rates of 4 and 0.4 μm/pulse for Pyrex glass and sapphire, respectively. The 266 nm pulsed laser (with a photon energy of 4.66 eV) requires only a two-photon excitation of electrons to the conduction band in Pyrex glass and sapphire. In contrast, the 355 nm laser (with a photon energy of 3.50 eV) necessitates a three-photon process at higher laser fluences, where nonlinear optical effects, including multiphoton processes, become significant. Additionally, the absorption coefficients for the 266 nm laser are higher in Pyrex glass (14.7 cm^−1^) and sapphire (5.19 cm^−1^) compared to the 355 nm laser, which has absorption coefficients of 1.93 cm^−1^ for Pyrex glass and 4.74 cm^−1^ for sapphire. Therefore, the 266 nm laser achieves a higher removal rate for both Pyrex glass and sapphire than the 355 nm laser. Based on these results, shorter UV wavelengths, like 266 nm, greatly enhance ablation efficiency and precision in glass micromachining by increasing absorption and reducing thermal damage.

Besides influencing ablation efficiency, the laser wavelength also affects the quality of the material removal process. A study on the precision of laser micromachining for Pyrex glass, using nanosecond-pulse Nd: YAG lasers at 266 and 355 nm, found that the 266 nm laser offered better precision [86]. This can be attributed to the ablation mechanisms, where photochemical ablation dominates at 266 nm, significantly reducing thermal effects such as warping, melting, thermal stress, residual stress, and rough surfaces, compared to the photothermal ablation process at 355 nm. The impact of shorter wavelengths (213 and 193 nm) on glass removal quality has also been examined and compared with 266 nm, using a 193 nm excimer laser operating at *t_p_* = 18 ns, and 266 and 213 nm Nd:YAG lasers at *t_p_* = 3.4 ns (*PRR* = 10 kHz, *d_L_* = 10 μm, and *F* = 21 J/cm^2^) [96]. The findings indicated that 193 and 213 nm pulses produced flat-bottomed profiles, while the 266 nm pulse resulted in spikes, likely due to particles redepositing within the crater. Based on these results, microfluidic channels’ ablation rates and precision can be effectively controlled by utilizing an optimal short wavelength with long laser pulses.

#### 3.1.6. Effects of Glass Type in Long-Pulse Micromachining

The type of glass significantly influences ablation efficiency, with factors such as chemical composition, melting point, and optical properties playing critical roles. For instance, fused silica exhibits different removal characteristics compared to soda–lime or borosilicate glasses due to its higher melting point and lower thermal expansion coefficient. Nieto et al. discussed material effects on ablation thresholds using Nd:YVO_4_ laser exposure (*t_p_* = 20 ns, *λ* = 1046 nm, and *d_L_* = 19 μm) [97]. The study revealed distinct threshold differences among soda–lime, borosilicate, fused silica, and sapphire, with values of 116 J/cm^2^, 954 J/cm^2^, 1054 J/cm^2^, and 1100 J/cm^2^, respectively. Lower thresholds lead to higher ablation efficiency, as less energy is needed to remove material, enabling faster and more effective material removal. Additionally, fused silica and borosilicate glass were tested using a CO_2_ laser micromachining system (*t_p_* = 20 μs, *λ* = 10.6 μm, *PRR* = 200 Hz, *d_L_* = 35 μm, and *P_p_* = 26 W) [93]. Under the same process parameters, the results demonstrated a higher removal rate on the surface of borosilicate glass, reaching up to 3.5 μm/pulse compared to 2 μm/pulse for fused silica. This difference is attributed to the lower ablation threshold of borosilicate glass relative to fused silica [97]. This reduction in threshold leads to the improved quality of the manufactured structures when a low fluence is applied. However, glasses with higher thresholds may exhibit better microchannel quality when a high fluence is utilized, as discussed next.

**Figure 6:**
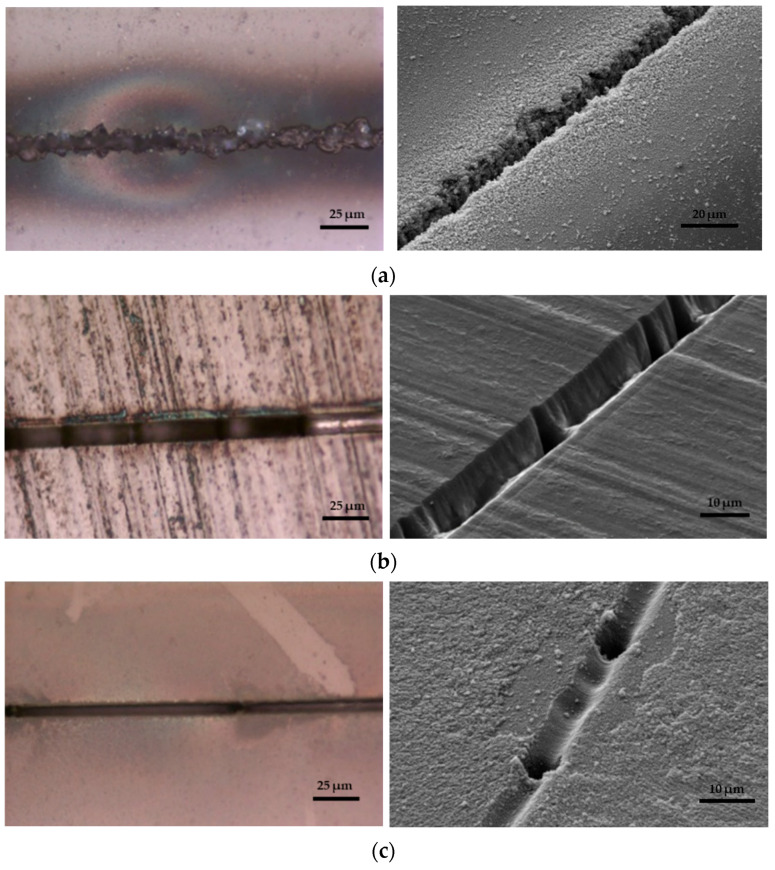
Optical micrographs (left) and SEM micrographs of microchannels in (**a**) fused silica glass, (**b**) diamond, and (**c**) sapphire, taken using a 1.3 ns Nd:YVO_4_ laser at *λ* = 355 nm, *PRR* = 5 kHz, *d_L_* = 1 μm, *V_scan_* = 100 mm/s, and *F* = 50 J/cm^2^ [65]. Adapted with permission from reference [65].

Different types of glass have been explored to understand the impact of glass materials on micromachining quality. A study by Perrone et al. utilized a CO_2_ laser (*λ* = 10.6 μm, *PRR* = 1000 Hz, and *d_L_* = 35 μm) to create microfluidic channels in various glass materials [98]. The results indicated that quartz microchannels (depth = 200 μm) likely exhibited smoother surfaces and fewer microcracks than other glass types, such as B270, Borofloat, Pyrex, and soda–lime glass. This is possible because quartz has a higher ablation threshold than other types, meaning it can endure higher laser intensities without damage. Glass materials like Borofloat and Pyrex may possess inherent variations or stress points that could result in less-uniform machining outcomes [99]. Another investigation employed an Nd:YVO_4_ laser (*t_p_* = 1.3 ns, *λ* = 355 nm, *PRR* = 5 kHz, *d_L_* = 1 μm, *V_scan_* = 100 mm/s, and *F* = 50 J/cm^2^) to ablate the surfaces of fused silica, diamond, and sapphire on a micrometer scale [65]. The experiment found that laser processing caused cracking and deformation in the fused silica glass, leading to unpredictable surface modifications that were difficult to replicate for microchannel fabrication (Figure 6a). In contrast, diamond and sapphire displayed only minor cracking and minimal distortion, as shown in Figure 6b,c. The variations in the results are likely due to their differing mechanical properties, such as density, elastic modulus, thermal conductivity, and optical properties [65]. Specifically, fused silica absorbs some 355 nm of light, while diamond and sapphire, with higher refractive indices, do not. Consequently, sapphire and diamond require more incident energy to reach the ablation threshold [86]. These studies demonstrate that the choice of material type plays a significant role when using long laser pulses to fabricate channels in microfluidic devices. These studies demonstrate that different glass types significantly impact long-pulse laser micromachining: glasses with lower ablation thresholds, like borosilicate, allow faster material removal but may produce rougher surfaces, while higher-threshold glasses, such as fused silica and quartz, yield smoother, higher-quality channels but require more energy for ablation. Ultimately, selecting the appropriate glass type is crucial for balancing efficiency and microchannel quality in laser-fabricated microfluidic devices.

#### 3.1.7. Summary of Reviewed Studies on Long-Pulse Laser Micromachining

The reviewed studies on long-pulse laser micromachining (pulse durations ≥ nanoseconds) highlight the complex interplay between ablation efficiency and quality, influenced by key parameters such as laser fluence, scanning speed, pulse duration, repetition rate, and wavelength, as well as the type of glass used, as summarized in Table 3. Generally, increasing laser fluence enhances material removal rates and etching depths but often at the expense of surface quality, leading to thermal damage, increased roughness, and microcracking. Similarly, slower scanning speeds and longer pulse durations can deepen ablation but exacerbate thermal effects, while higher repetition rates boost efficiency through heat accumulation but risk compromising quality due to thermal stress. Conversely, shorter wavelengths tend to improve both efficiency and precision by leveraging photochemical mechanisms over photothermal ones. The choice of glass type further modulates these outcomes, with materials like soda–lime glass exhibiting lower ablation thresholds for higher efficiency, whereas quartz and fused silica offer better quality due to their higher resistance to thermal damage. These findings underscore the necessity for meticulous parameter optimization to achieve the desired balance between efficiency and quality in glass microfluidic fabrication using long-pulse lasers.

### 3.2. Short-Pulse Laser Micromachining of Glass

Recent studies have shown that short laser pulses (picosecond) are highly effective for fabricating glass microfluidic channels due to their distinct material removal mechanisms [100,101,102]. The ablation process is influenced by key parameters, such as the absorption coefficient (α) and thermal diffusivity (κ) of the material, which determine the thermal relaxation time—the period required for a material to dissipate approximately 63% of the incident thermal energy [103,104]. Since picosecond laser pulses are significantly shorter than the thermal relaxation time of glass (which operates in the nanosecond range), the ablation process occurs before substantial heat diffusion takes place, thereby minimizing thermal effects and reducing the HAZ. This allows nonlinear absorption processes to dominate the removal mechanism [26,105]. Several reviews in the literature have explored the fundamental mechanisms of picosecond-pulse laser interactions with matter [106,107]. The ability of picosecond-pulse lasers to minimize thermal effects and reduce the HAZ is critical for achieving high-quality material removal, directly impacting the precision needed for microfluidic device fabrication [11,108]. Therefore, understanding and optimizing etching efficiency and quality is essential for a more cost-effective manufacturing process, ensuring precise and smooth microchannels while maintaining the functionality of glass-based microfluidic devices [7]. Several key processing parameters affect the efficiency and quality of short-pulse laser ablation, including fluence, scanning speed, pulse duration, repetition rate, wavelength, and the type of glass used [109,110].

#### 3.2.1. Impact of Fluence on Short-Pulse Micromachining

Several studies have investigated the effect of short-pulse fluence on the efficiency of glass ablation, highlighting the significance of fluence in determining material removal depth and rate. Fan et al. conducted an in-depth study of the effect of fluence on the depth of soda–lime microfluidic channels, utilizing a picosecond laser (*t_p_* = 10 ps, *λ* = 355 nm, *PRR* up to 800 kHz, and *d_L_* = 13.8 μm) [100]. The average crater depth increased from 0.035 to 0.143 μm as the laser fluence ranged from 3.28 to 6.49 J/cm^2^, with more energy available for material removal. A broader fluence range (2 to 12 J/cm^2^) was examined using a picosecond laser (*t_p_* = 10 ps, *λ* = 355 nm, *PRR* = 800 kHz, *d_L_* = 15 μm, and *V_scan_* = 200 mm/s) to assess the borosilicate etching efficiency [111]. The results indicated a significant increase in the efficiency (from 0.1 to 3.2 μm^3^/μJ) as the fluence rose from 2 to 3 J/cm^2^, after which the efficiency stabilized around 3.2 μm^3^/μJ for fluence values exceeding 3 J/cm^2^. A similar approach was noted when the fluence varied from 5.5 to 23.5 J/cm^2^ using a picosecond laser (*t_p_* = 13 ps, *λ* = 1064 nm, *PRR* = 100 kHz, *d_L_* = 27 μm, *V_scan_* = 2 m/s) on borosilicate glass [112]. The ablation efficiency rose from 0.9 to 1.8 μm^3^/μJ as the fluence increased from 5.5 to 12 J/cm^2^, then it stabilized at approximately 1.6 μm^3^/μJ for fluences above 12 J/cm^2^. This can be attributed to the formation of laser-induced plasma on the material surface at high fluence [113]. The plasma serves as a shield, reflecting or scattering laser energy away from the removal area, reducing the energy available for material removal. This phenomenon, referred to as plasma shielding, results in decreased efficiency despite the higher fluence applied. A similar trend in stabilizing the ablation efficiency was observed when higher laser fluences, ranging from 10 to 37 J/cm^2^, were applied for thin glass ablation using a thin-disk laser (*t_p_* = 6 ps, *λ* = 355 nm, *PRR* = 20 kHz, *d_L_* = 10 μm, and *V_scan_* = 100 mm/s) [114]. The results demonstrated an increase in the effective cutting speed from 5 mm/s to 19 mm/s as the fluence increased from 10 to 15 J/cm^2^, while no significant improvement was noted beyond 15 J/cm^2^. These findings illustrate that applying the appropriate fluence before reaching the stabilization state is crucial to prevent energy deposition in areas surrounding the ablation zone, which may impact the quality of laser microchannel processing in glass.

At optimal fluence levels, a higher ablation quality is achieved through precise material removal and minimal thermal damage, which may result in smoother and more accurate features. Several studies have examined the impact of laser fluence on etching quality, mainly focusing on surface roughness. Wlodarczyk et al. investigated the influence of fluence on the surface roughness of borosilicate using a picosecond-pulse laser (*t_p_* = 6 ps, *λ* = 515 nm, *PRR* = 100 kHz, *d_L_* = 21 μm, and *V_scan_* = 150 mm/s), finding a slight increase in the average surface roughness from 1.55 µm to 1.72 µm as the fluence increased from 16 to 33 J/cm^2^ [115]. In a related study, the same group examined the etching quality of Borofloat 33 glass using a picosecond-pulse laser (*t_p_* = 6 ps, *λ* = 515 nm, *PRR* = 20 kHz, *d_L_* = 24 μm, and *V_scan_* = 40 mm/s), observing that surface roughness remained around 1.6 µm, with minimal variation across a range of fluences from 11 to 31 J/cm^2^ [14]. A further investigation into the influence of fluence on the ablation quality of AF32 Eco thin glass was conducted using a thin-disk laser (*t_p_* = 6 ps, *λ* = 343 nm, *PRR* = 20 kHz, *d_L_* = 10 μm, and *V_scan_* = 100 mm/s), with fluence values ranging from 20 to 37 J/cm^2^ [114]. The results indicated that a small HAZ (<20 μm) appeared around the holes produced at all the fluence values, with no microcracks observed and only the partial redeposition of material, suggesting that increasing the fluence did not significantly affect the removal quality within this range. Additionally, the effect of low laser fluences of 1.41 and 2.83 J/cm^2^ was studied using picosecond lasers (*t_p_* = 10 ps, *λ* = 1064 nm, *PRR* = 200 kHz, *d_L_* = 60 μm, and *V_scan_* = 5 mm/s) for microgroove fabrication on single-crystal diamond [79]. At both fluences, cracks and chips appeared at the edges of the microgrooves, with a small amount of ablation debris (325 nm in size) generated at 1.41 J/cm^2^, while at 2.83 J/cm^2^, the microgroove was produced without any debris. Therefore, at the optimal laser fluence, glass micromachining achieves a higher ablation quality with precise material removal and minimal thermal damage, resulting in smoother and more accurate features, while variations in fluence within a certain range have little effect on surface roughness or removal quality.

#### 3.2.2. Impact of Scanning Speed on Short-Pulse Micromachining

The scanning speed of a picosecond laser significantly affects ablation efficiency by influencing the interaction time between laser pulses and the material and the overlap between consecutive pulses. Markauskas and colleagues investigated the impact of scanning speeds ranging from 200 to 1000 mm/s on ablation efficiency using a picosecond laser (*t_p_* = 10 ps, *λ* = 355 nm, *PRR* = 653 kHz, *d_L_* = 15 μm, and *P_avg_* = 2.1 W) [111]. The experiments revealed that the optimal laser scanning speed for borosilicate glass was 800 mm/s, achieving 5.75 μm^3^/μJ of etching efficiency, compared to 3.25 and 4 μm^3^/μJ at 200 and 1000 mm/s, respectively. Performance decreases at lower scanning speeds (<800 mm/s) due to the increased shielding of the laser beam by plasma and debris resulting from heat accumulation. Conversely, at scanning speeds above 800 mm/s, the loss in efficiency is attributed to a decrease in the temperature of the removal zone, as the increasing distance between the laser pulses reduces the energy concentration, preventing the material from reaching the optimal temperature for effective ablation [116].

**Figure 7:**
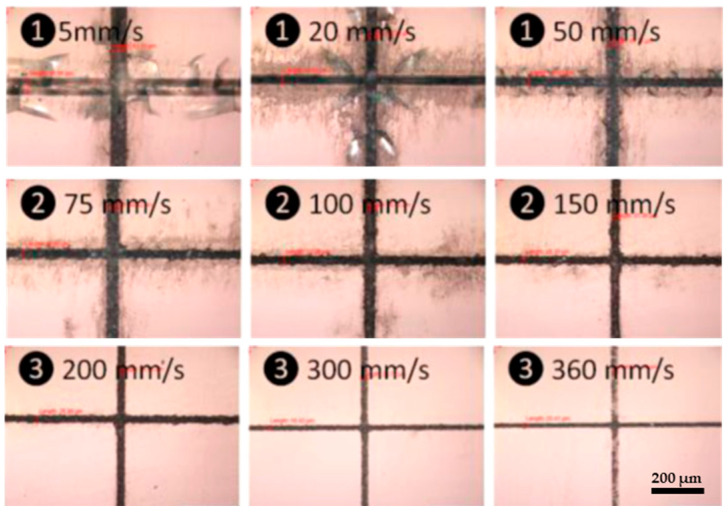
Optical images of cross-ablated glass captured during a single pass at varying scanning speeds (5–360 mm/s) using a 1064 nm laser with a pulse duration of less than 12 ps, a pulse frequency of 50 kHz, a laser fluence of 9.0 J/cm^2^, and a spot size of approximately 25 μm [117]. Adapted with permission from reference [117].

In addition to the impact of scanning speed on ablation efficiency, speed also affects removal quality, as reported by Wang et al. [117]. They used a picosecond laser (*t_p_* = <12 ps, *λ* = 1064 nm, *PRR* = 50 kHz, *d_L_* = 25 μm, and *F* = 9.0 J/cm^2^) with scanning speeds ranging from 5 to 360 mm/s. The results indicate three distinct regions: at lower speeds (below 75 mm/s), cracks and debris are produced on the glass surface; at medium speeds (75–150 mm/s), cracks disappear, and primarily debris remains; and at higher speeds (above 150 mm/s), neither cracks nor debris are produced, as shown in Figure 7. This may be because, at lower scanning speeds, more pulses are delivered to a single spot, leading to a greater overlap of laser spots. This leads to more intense shockwaves and greater heat generation due to the prolonged interaction between the laser and the glass [50,117]. For instance, at scanning speeds below 75 mm/s, over 16 pulses can impact each spot, whereas at higher scanning speeds above 200 mm/s, fewer than 6 pulses are delivered. As a result, the extended exposure at lower speeds causes thermal cracks and leads to debris deposition as droplets in the etching area, negatively affecting surface quality. The optimal scanning speed for laser ablation of glass, without generating cracks or debris, was found to be above 200 mm/s. However, it is important to note that a significant increase in scanning speed may lead to the formation of very shallow microchannels, which are unsuitable for microfluidic devices that require high aspect ratio microchannels. This limitation can be addressed by increasing the number of laser passes over the removal area [118,119]. Eventually, optimal scanning speeds in short-pulse laser micromachining maximize the ablation efficiency and produce high-quality, crack-free microchannels, while speeds that are too low cause cracks and debris due to heat buildup, and speeds that are too high reduce the removal depth. Therefore, selecting the right scanning speed is crucial for the efficient and precise microfabrication of glass microfluidic devices.

#### 3.2.3. Impact of Pulse Duration on Short-Pulse Micromachining

Research has shown that shorter pulse durations enhance glass ablation efficiency by delivering high peak power within extremely brief time intervals, resulting in higher removal rates than longer pulses in the same short-duration range. The effect of picosecond pulses on the BK7 glass ablation rate was investigated using a mode-locked Yb-Fiber laser (*λ* = 1035 nm, *PRR* = 250 kHz, *d_L_* = 20 μm, and *F* = 3.2 J/cm^2^) [120]. The study demonstrated a 37% increase in the etching rate when applying shorter pulses of 1.5 ps compared to 10 ps. Further investigation at a different laser wavelength (517 nm) and fluence (4 J/cm^2^) examined the effect of pulse duration (14 and 1.5 ps) on glass ablation efficiency [120]. The results revealed that the shorter pulse duration of 1.5 ps yielded a higher ablation rate of 0.65 mm^3^/(W·min) compared to 0.48 mm^3^/(W·min) for 14 ps. A clear impact of pulse duration on process efficiency was also observed using a 345 nm wavelength and a fluence of 1.8 J/cm^2^, where the rate was significantly higher at 1.5 ps (0.50 mm^3^/(W·min)) compared to 10 ps (0.10 mm^3^/(W·min)). The increased etching efficiency with shorter pulses can be attributed to the greater peak power intensity (W/cm^2^) associated with these pulses. Shorter pulses possess higher intensity, enabling them to break atomic bonds more effectively [121]. Additionally, the lower ablation threshold of shorter pulses (1.5 ps), where energy dissipation is minimal, compared to longer pulses (>10 ps), also contributes to the improved etching efficiency. The removal mechanism of shorter pulses (<5 ps) further enhances process effectiveness, as laser removal is primarily driven by multiphoton and avalanche ionization. However, near 5 ps, the removal mechanism shifts, with thermal dissipation processes becoming predominant [122,123].

The benefits of shorter pulse durations on the ablation rate also positively influence removal precision, as reported in the study by Ly et al. [124]. This study utilized a tunable laser system with pulse durations ranging from 0.4 to 60 ps (*λ* = 1053 nm, *PRR* = 10 Hz, *d_L_* = 50 μm, and *F* = up to 52.7 J/cm^2^) to investigate the damage morphology in fused silica. The results indicated that a homogeneous etching process resulted in smooth craters, with surface roughness primarily stemming from the redeposition of ablated material. For pulses as short as 1.8 ps, smooth craters were noted, whereas longer pulse durations showed damage morphologies with local defects or areas more vulnerable to damage. The surface roughness increased with longer pulse durations; the roughness grew from 40 nm to 320 nm as the pulse width rose from 1.8 ps to 59 ps. The experiments suggest that decreasing the pulse duration positively affects the removal rate and the ablation quality. Therefore, compressing the pulse in picosecond-pulse laser systems proves to be more effective for glass micromachining.

#### 3.2.4. Impact of Repetition Rate on Short-Pulse Micromachining

The effect of the repetition rate of picosecond lasers on ablation efficiency is a subject of considerable interest due to its implications for speed and precision in material processing. Research on glass using a picosecond laser (*t_p_* = <10 ps, *λ* = 1 μm, *d_L_* = 25 μm, and pulse energy = 10 μJ) demonstrated that increasing the repetition rate from 500 to 2000 kHz significantly enhanced the removal rate, reaching 14 mm^3^/min at 2000 kHz compared to only 2 mm^3^/min at 500 kHz, which led to an increase in the microchannel depth [125]. This rate increase was attributed to a reduction in the ablation threshold as the repetition rate increased. However, Karimelahi et al. reported a decrease in depth with an increasing repetition rate from 200 to 1000 kHz using an Nd:YVO_4_ laser (*t_p_* = 12 ps, *λ* = 532 nm, *d_L_* = 3.4 μm, *V_scan_* = 100 μm/s, and pulse energy = 12 μJ) on fused silica material [126]. The depths were measured as 1 mm, 500 µm, and 200 µm at 200, 400, and 1000 kHz, respectively, with a large HAZ (greater than 100 µm at 400 kHz and 200 µm at 1000 kHz) surrounding the entrance, while no HAZ was observed at 200 kHz. Crimella et al. noted the same approach using a ps laser system (*t_p_* = 10 ps, *λ* = 1064 nm, *d_L_* = 26.5 μm, *V_scan_* = 500 cm/s, and pulse energy = 360 μJ) on soda–lime glass [66]. Increasing the pulse repetition rate to 800 kHz resulted in a 70% reduction in cutting efficiency compared to 400 kHz. The findings indicate that while high repetition rates can enhance ablation efficiency due to heat accumulation, there is a threshold where efficiency may decrease because of plasma or debris shielding effects.

**Figure 8:**
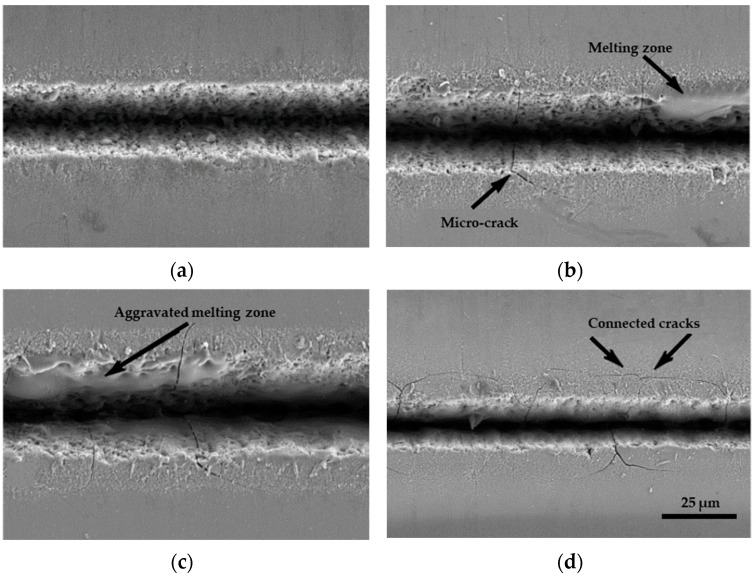
SEM images of microchannels formed on the soda–lime glass by high-repetition-rate, short-pulse micromachining: (**a**) fluence (*F*) = 55.76 J/cm^2^ (500 kHz); (**b**) *F* = 66.91 J/cm^2^ (600 kHz); (**c**) *F* = 78.06 J/cm^2^ (700 kHz); (**d**) *F* = 89.22 J/cm^2^ (800 kHz) [100]. Adapted with permission from reference [100].

Higher repetition rates can enhance material removal rates but may also lead to increased thermal damage and surface roughness. Several studies have examined the effect of repetition rate on removal quality. The influence of repetition rate on the quality of laser cuts in borosilicate glass was investigated using a picosecond laser (*t_p_* = 13 ps, *λ* = 1064 nm, *d_L_* = 27 μm, *V_scan_* = 100 cm/s, and *F* = up to 24.4 J/cm^2^) [110]. The experiment demonstrated that heat accumulation effects became more pronounced as the pulse repetition rate increased from 400 to 650 kHz. At 600 kHz, the cut edge exhibited slight melting, while at 650 kHz, melting was more significant. Cracking and chipping along the cut line were observed up to 600 kHz, with fracture and chip sizes on the front side remaining relatively consistent (7.4 ± 2.7 μm), unaffected by the pulse repetition rate. Band-like damage was evident on both sides of the cut. At lower repetition rates, they appeared as separate spots, while at higher rates, they merged into deeper cracks and chips. The increased roughness of the cut wall could negatively impact the strength of the workpiece. The findings indicated a significant increase in cut wall roughness from 1.8 μm to 32 μm as the pulse repetition rate rose from 400 to 600 kHz. The formation of remelted, chin-like structures at the bottom of the cuts, which developed rapidly with the increasing repetition rate, was also noted. Furthermore, Fan et al. observed the formation of microcracks and localized melting along the edges of microchannels in soda–lime glass when using a picosecond laser micromachining system (*t_p_* = 10 ps, *λ* = 355 nm, *d_L_* = 13.6 μm, *V_scan_* = 400 cm/s, and *F* = up to 89.22 J/cm^2^) at higher repetition rates (600–800 kHz) compared to 500 kHz (Figure 8) [100]. The irregular edges were likely due to excessive heat accumulation during the ablation process at high repetition rates [127]. In general, increasing the repetition rate in short-pulse laser micromachining can initially enhance the material removal rate and microchannel depth. However, excessively high rates lead to heat accumulation, plasma or debris shielding, and greater thermal damage, ultimately reducing the ablation efficiency and the surface quality. Therefore, while higher repetition rates can speed up processing, they must be carefully optimized to avoid increased surface roughness, melting, and microcracking in glass microfluidic devices.

#### 3.2.5. Impact of Laser Wavelength on Short-Pulse Micromachining

Different wavelengths interact with glass in distinct ways because of variations in absorption, as Wlodarczyk and colleagues reported when examining the effect of wavelengths on AF32 glass ablation efficiency using a thin-disk laser (*t_p_* = 6 ps, *PRR* = 400 kHz, *d_L_* = 22.5–10 μm, and *V_scan_* = 2 m/s) at three wavelengths: 1030, 515, and 343 nm [114]. The results demonstrated that, at the same fluence of 20 J/cm^2^, the highest cutting speed (110 mm/s) was attained with the 1030 nm wavelength, compared to 70 mm/s with 515 nm and 30 mm/s with 343 nm. A further analysis of the effect of wavelength (1035, 517, and 345 nm) on BK7 glass processing efficiency was conducted using a mode-locked Yb-Fiber laser (*t_p_* = 5 ps, *PRR* = 250 kHz and 500 kHz, *d_L_* = 25–12 μm, and *F* = 1.8 J/cm^2^) [120]. The findings revealed that the highest ablation rate, 0.25 mm^3^/(W·min), occurred at 1035 nm, in contrast to 0.21 mm^3^/(W·min) at 517 nm and 0.15 mm^3^/(W·min) at 343 nm. The ablation efficiency was shown to depend heavily on the wavelength, with the largest removal rate achieved at the longer wavelength. This may be attributed to the optical properties of glass materials, as the absorption coefficient decreases notably with longer wavelengths, permitting the laser beam to penetrate deeper into the material and providing a more uniform energy distribution within the glass [128,129].

Regarding the quality of glass micromachining, several studies have examined how wavelength affects picosecond-pulse ablation. The findings indicate that shorter wavelengths generally result in higher absorption, allowing for a more precise process and reduced surface roughness [130]. For example, one study explored the effects of wavelength on the removal quality of thin glass plates using a laser operating at 1030, 515, and 343 nm (*t_p_* = 6 ps, *PRR* = 20 kHz, *d_L_* = 22.5–10 μm, and *V_scan_* = 100 mm/s) [114]. The results showed that at 1030 nm, the cutting quality was the worst, with debris, redeposited material, and occasional microcracks observed within the HAZ, which was approximately 30 μm wide. At 515 nm, satisfactory cutting results were achieved, with the laser-cut edges free of microcracks or chipping and a HAZ of less than 20 μm. The best cutting quality was observed at 343 nm, where the HAZ remained under 20 μm, with no microcracks or chipping present. The improvement in removal quality can be attributed to the fact that shorter wavelengths result in a slower removal rate, offering greater precision and better control over the etching process, thereby enhancing the removal quality. Consequently, considering the effects of wavelength, a short-UV-wavelength ps laser is preferable for fabricating high-quality microchannels, keeping in mind that the fabrication process may take longer.

#### 3.2.6. Impact of Glass Type on Short-Pulse Micromachining

The type of glass significantly influences the efficiency of picosecond laser ablation, as different materials possess varying thermal properties, absorption characteristics, and chemical compositions. These factors impact the laser and glass interaction, resulting in different etching rates. Various glass materials have been examined to evaluate the effect of glass type on ablation efficiency, particularly concerning the ablation threshold, using a thin-crystal-disk laser system (*t_p_* = 10 ps, *λ* = 1030 nm, and *d_L_* = 10–19 μm) [97]. The thresholds of soda–lime glass, fused silica, sapphire, and borosilicate glass have been analyzed as indicators of ablation efficiency, demonstrating an evident reliance on the type of glass. The results showed that soda–lime and borosilicate glasses exhibited lower ablation thresholds of 9.54 and 9.40 J/cm^2^, respectively, compared to fused silica (11.02 J/cm^2^) and sapphire (13.05 J/cm^2^). This suggests that soda–lime and borosilicate glass display greater removal effectiveness than fused silica and sapphire at the same applied fluence [131].

The type of glass significantly impacts the quality of picosecond-pulse laser ablation, as different glasses exhibit distinct optical properties, thermal conductivities, and absorption rates. The effects of soda–lime glass, fused silica, sapphire, and borosilicate on removal quality were investigated using a picosecond laser system operating at a 1030 nm wavelength and a 10 ps pulse width [97]. The results indicate that, except for sapphire, all the glasses displayed concentric rings due to interference between the incident waves and the reflected waves of the shockwave front. This phenomenon occurs because the shock front creates a high-quality optical surface, allowing coherent interference between reflected and unreflected beams, resulting in concentric interference rings that extend to considerable radii. In contrast, sapphire exhibited a different morphology in the ablated areas, with a raised rim surrounding smooth craters. This suggests the presence of a very thin melt zone, just a few micrometers thick, during the ablation process, where molten material moves from the center to the edges of the crater, forming a thin rim around the ablated area. Overall, the type of glass plays a key role in short-pulse laser micromachining, with soda–lime and borosilicate glass showing lower ablation thresholds and higher ablation efficiency than fused silica and sapphire. Each glass type also produces distinct surface features after ablation, making material selection crucial for optimizing both efficiency and microchannel quality.

#### 3.2.7. Summary of Reviewed Studies of Short-Pulse Laser Micromachining

The reviewed studies on short-pulse laser micromachining demonstrate a delicate balance between ablation efficiency and quality, influenced by parameters such as fluence, scanning speed, pulse duration, repetition rate, wavelength, and the type of glass, as summarized in Table 4. Research indicates that increasing fluence generally enhances ablation depth and material removal rates, but it can introduce surface defects and reduce quality beyond certain thresholds. Scanning speed exhibits an optimal range where efficiency is maximized; deviations from this range result in either excessive heat accumulation or insufficient energy deposition. Shorter pulse durations are particularly advantageous, as they concentrate energy delivery, thereby increasing ablation rates while minimizing thermal diffusion and associated damage, leading to smoother surfaces. Higher repetition rates can boost overall removal rates but may induce thermal stress and surface irregularities if not carefully managed. Wavelength selection also plays a critical role, with longer wavelengths facilitating higher cutting speeds and deeper penetration, whereas shorter wavelengths offer improved precision and reduced HAZs. Additionally, the choice of glass type significantly impacts both efficiency and quality, with variations in ablation thresholds and thermal properties dictating the optimal processing conditions. These findings underscore the importance of precise parameter tuning in picosecond laser micromachining to achieve the desired outcomes for glass microfluidic applications.

### 3.3. Ultrashort-Pulse Laser Micromachining of Glass

A fundamental understanding of ultrashort-pulse laser ablation is essential for using laser systems to fabricate glass microchannels. When femtosecond-pulse lasers interact with glass, they excite electrons from the valence band to the conduction band through a multiphoton absorption process [106]. Due to the nonlinear nature of this absorption mechanism, a transparent substrate efficiently absorbs tightly focused, ultrashort laser pulses [132]. At extremely high photon densities (~10^12^ W/cm^2^), energy is deposited into the substrate on a femtosecond (10^−15^ s) timescale, which is much shorter than both the heat diffusion time (on the nanosecond scale) and the electron-cooling rate (on the order of 1 ps) [133,134]. This results in a process known as “cold ablation”, which is characterized by minimal thermal damage and fewer cracks in the surrounding material compared to long-pulse laser ablation [135,136]. Several literature reviews have discussed the fundamental mechanisms of femtosecond-pulse laser interactions with matter [137,138]. The ability of femtosecond-pulse lasers to minimize thermal effects and reduce the HAZ is crucial for achieving high-quality material removal, directly influencing the precision required to fabricate glass-based microfluidic devices [29]. Therefore, understanding and optimizing ablation efficiency and quality by exploring the effects of key processing parameters—such as fluence, scanning speed, pulse duration, repetition rate, and wavelength—will help achieve the precise microchannels needed for high-performance glass fluidic devices [139,140].

#### 3.3.1. Effects of Laser Fluence in Ultrashort-Pulse Micromachining

The ultrashort laser fluence is fundamental in determining the efficiency of glass removal. Generally, higher fluence leads to quicker material removal and deeper channels. Xu et al. reported fabricating craters on borosilicate glass using a Ti:sapphire laser system (*t_p_* = 35 fs, *λ* = 800 nm, *PRR* = 10 Hz, and *d_L_* = 27 μm [141]). The ablation rate increased from 0.19 to 0.63 μm/pulse as the fluence rose from 0.9 to 3.5 J/cm^2^, stabilizing at 0.65 μm/pulse for fluences between 3.5 and 11 J/cm^2^. This stabilization at higher fluences was attributed to material plasma shielding, which absorbs, reflects, or scatters part of the incident laser energy, preventing additional energy from reaching the substrate. Macernyte et al. investigated the effects of varying energy densities on the depth of soda–lime microchannels using a Yb:KGW laser (*t_p_* = 280 fs, *λ* = 1030 nm, *PRR* = 60 kHz, *d_L_* = 20 μm, and *V_scan_* = 100 mm/s) [142]. The results demonstrated the fluence’s influence on depth, with the groove depth increasing from 55 to 90 µm as the fluence rose from 9.5 to 23.5 J/cm^2^. The study also explored how different ablation conditions (dry, water, KOH, and NaCl aqueous solutions) affected microchannel depth at varied fluence levels. In all the conditions, an increase in depth was noted with higher fluence. However, the deepest channels were produced when no solution layer was applied, likely due to the partial absorption and scattering of the laser beam by the liquid layer, as reported in our previous work [143]. Additionally, the impact of a very high fluence (up to 1958 J/cm^2^) on the fused silica etching depth was studied using a femtosecond laser (*t_p_* = 190 fs, *λ* = 1030 nm, *PRR* = 1 kHz, *d_L_* = 7.9 μm, and *V_scan_* = 100 mm/s) [144]. The experiments indicate that channels deeper than 850 µm were directly drilled when the fluence increased to 1958 J/cm^2^, compared to just 150 µm at 244 J/cm^2^. This significant increase in the channel depth suggests that femtosecond laser direct drilling in air can yield effective results without any additional setup. The increased depth was attributed to the higher kinetic energy of the ablated material at elevated laser fluences [145], enhancing the ability of glass to be ejected and resulting in deeper channels. The findings underscore that increasing laser fluence initially leads to faster material removal and deeper channels, but beyond a certain point, efficiency plateaus due to plasma shielding effects.

Laser fluence is a key factor in the quality of glass microfabrication, influencing the formation of ablation features. Several studies have examined how varying laser fluence affects glass micromachining, emphasizing its impact on factors like surface morphology. A study by Xiao and Xin investigated drilling microchannels into fused silica using a Yb:KGW femtosecond laser (*t_p_* = 190 fs, *λ* = 1030 nm, *PRR* = 1 kHz, *d_L_* = 7.9 μm, and *V_scan_* = 200 µm/s) with varying fluences (244–1958 J/cm^2^) [144]. The results indicated that at 1101.7 J/cm^2^ and lower fluences, smooth sidewalls were achieved, while higher fluences, such as 1958 J/cm^2^, resulted in rougher sidewalls. Surface roughness (Ra) was measured at fluences of 734, 1101, and 1958 J/cm^2^, yielding values of 0.65, 0.66, and 5.17 µm, respectively, showing a significant increase with higher fluences. This rise in roughness can be attributed to increased challenges in debris removal at higher fluences, causing ablated material to redeposit on the sidewalls [145]. Additionally, cracks were noted around the solid parts of the microchannels at higher fluences, likely due to the stress induced by rapid heating and material confinement during laser irradiation. This stress becomes sufficient at a high fluence to fracture the material and cause cracking. Furthermore, the effect of fluence on the ablation quality of fused silica at lower fluences (5 to 22 J/cm^2^) was investigated using an ultrashort-pulse laser (*t_p_* = 220 fs, *λ* = 343 nm, *PRR* = 50 kHz, *d_L_* = 14.5 μm, and *V_scan_* = 100 mm/s) [146]. The experiments revealed no significant topological changes or chipping at the cavity edges as the fluence increased from 5 to 22 J/cm^2^. However, higher fluences resulted in molten particles redepositing on the surface, increasing surface roughness. At a fluence of 5 J/cm^2^, the surface roughness was 0.37 µm, while at 22 J/cm^2^, it rose to 0.54 µm. These findings underscore the impact of high fluence on removal quality, such as increasing surface roughness and the risk of cracks from thermal stress. Thus, optimizing fluence is crucial for producing high-quality microfluidic channels on glass substrates.

#### 3.3.2. Effects of Scanning Speed in Ultrashort-Pulse Micromachining

The scanning speed of an ultrashort laser is crucial for fabricating glass microchannels. The effect of scanning speed on the femtosecond laser ablation of soda–lime glass was examined using a Yb:KGW laser (*t_p_* = 280 fs, *λ* = 1030 nm, *PRR* = 60 kHz, *d_L_* = 20 μm, and *P_avg_* = 5 W) [142]. The findings indicated that the groove depth in the glass decreased to 40 µm as the scanning speed increased to 300 mm/s, compared to 135 µm when the speed was reduced to 50 mm/s. This reduction in groove depth is associated with the lower overlap of laser pulses at higher scanning speeds, which may decrease the ablation depth. In a more recent study, the impact of low scanning speeds (20–500 μm/s) on the channel depth of fused silica was investigated using a Yb:KGW laser (*t_p_* = 190 fs, *λ* = 1030 nm, *PRR* = 1 kHz, *d_L_* = 7.9 μm, and *F* = 734 J/cm^2^) [144]. The method involved drilling channels by positioning the focal spot beneath the rear surface of the sample and moving the sample downward until the front surface reached the focal point. The results revealed that as the sample movement speed increased, the channel depth initially grew, peaking at an optimal speed before declining at higher speeds. For example, the channel depth rose from 400 to 750 µm as the scanning speed increased from 20 to 150 μm/s, but then fell to 250 µm as the speed increased further to 500 μm/s. This pattern can be explained by the concept that continuous material removal at lower speeds requires the material to maintain high transparency along the beam propagation path. However, prolonged laser irradiation can damage the material in front of the channel at excessively low scanning speeds, leading to a loss of transparency in the glass. The non-transparent region obstructs the laser beam, disrupting the removal process. Consequently, slower scanning speeds result in more significant laser irradiation, causing early laser shielding and reduced channel depths.

In addition to the effect of scanning speed on the depth of microchannels using femtosecond pulses, several studies have explored its impact on removal quality. For instance, one study by Zhang et al. used a Ti:sapphire femtosecond laser (*t_p_* = 100 fs, *λ* = 800 nm, *PRR* = 1 kHz, and *P_avg_* = 50 mW) to fabricate microchannels in quartz glass [24]. The study found that the ablation quality improved at lower scanning speeds (<1.5 mm/s), producing smooth microgrooves. However, heat accumulation at speeds below 0.5 mm/s led to tiny cracks forming on the workpiece. The etching quality deteriorated at higher scanning speeds (>1.5 mm/s), with the surface losing straightness and the microgroove becoming rough. According to the research findings, to fabricate microfluidic channels on glass, the scanning velocity should be optimized to prevent heat accumulation due to excessively low scanning speeds and avoid channels’ discontinuous appearance at elevated scanning velocities. More recently, Liao et al. investigated the influence of femtosecond laser scanning strategies on the surface roughness of rectangular microchannels in silica glass [147]. The study demonstrated that the scanning path and sequence significantly affected the microchannels’ shape, size, and roughness. By carefully adjusting the scanning strategy, they achieved rectangular microchannels with an average sidewall taper angle of less than 5° and a surface roughness of 2.53 µm. Ultimately, in the ultrashort-pulse laser micromachining of glass, lower scanning speeds increase channel depth but risk heat buildup and cracking, while higher speeds reduce depth and can cause rough, uneven channels—making optimal speed and scanning strategy essential for achieving deep, smooth microchannels.

#### 3.3.3. Effects of Pulse Duration in Ultrashort-Pulse Micromachining

The pulse duration of ultrashort lasers significantly affects ablation efficiency. Shorter pulse durations (in the femtosecond range) allow for more precise energy deposition, which minimizes thermal diffusion and HAZs, potentially enhancing process effectiveness. Xu et al. examined the impact of reducing the pulse duration from 500 fs to 35 fs on the ablation efficiency of borosilicate glass using a Ti:sapphire laser (*λ* = 800 nm, *PRR* = 10 Hz, and *d_L_* = 27 μm) [141]. At the same fluence (3.5 J/cm^2^), the etching rate for the 500 fs pulse laser was measured at 0.22 μm/pulse, compared to 0.65 μm/pulse for the 35 fs pulses. This difference is attributed to the lower ablation threshold at shorter pulse durations, with thresholds of 0.42 J/cm^2^ for 35 fs and 2.1 J/cm^2^ for 500 fs. The findings suggest that multiphoton absorption is the primary mechanism for crater formation during 35 fs pulse ablation, while impact ionization plays a critical role in generating conduction band electrons during 500 fs pulse ablation [148].

In addition to examining the impact of shorter femtosecond-pulse lasers on the ablation rate, the Xu et al. study also investigated the laser’s effect on removal quality using 35 fs and 500 fs lasers (*λ* = 800 nm, *PRR* = 10 Hz, and *d_L_* = 27 μm) to fabricate craters on borosilicate glass [141]. The results demonstrate the influence of pulse duration. For the 35 fs pulses, a smooth crater was formed, surrounded by a rippled zone, with no re-solidified molten material at the rim. In contrast, the 500 fs pulses produced molten material and a rough surface with periodic structures, cracks, and/or ripples. The decrease in quality is due to the greater beam penetration depth at longer pulse durations, which promotes mechanical relaxation processes in the glass, resulting in the formation of laser-induced periodic surface structures [149]. Conversely, shorter pulse durations enable efficient multiphoton absorption, reducing the beam penetration depth and yielding smoother ablated craters. Therefore, shorter pulse durations in the ultrashort-pulse laser micromachining of glass greatly improve ablation efficiency and quality, producing higher removal rates and smoother surfaces by minimizing thermal effects. This makes ultrashort pulses ideal for fabricating precise, crack-free microchannels in glass microfluidic devices.

#### 3.3.4. Effects of Repetition Rate in Ultrashort-Pulse Micromachining

The repetition rate of ultrashort lasers significantly influences ablation efficiency during glass processing, primarily due to heat accumulation and incubation effects. As the repetition rate increases, there is less time for heat to dissipate between pulses, resulting in a cumulative thermal effect. This can lower the ablation threshold and enhance the material removal efficacy. A study by Lopez et al. on micromachining soda–lime glass using a Yb-doped laser (*t_p_* = 500 fs, *λ* = 1030 nm, *d_L_* = 3.8 μm, and *F* = 112 J/cm^2^) at repetition rates ranging from 5 Hz to 200 kHz revealed that the etching depth increased from 147 to 350 µm as the repetition rate rose from 5 Hz to 20 kHz [150]. However, beyond 20 kHz, the depth began to decrease, and a HAZ appeared at the surface due to overheating. This occurred because the time between consecutive pulses was significantly shortened, potentially leading to material damage and plasma shielding. These findings indicate that heat accumulation is beneficial up to 20 kHz but becomes harmful beyond that point. The effect of repetition rate (10, 100, and 500 Hz) on the ablation rate was also examined in borosilicate glass using a Ti:sapphire laser (*t_p_* = 35 fs, *λ* = 800 nm, *d_L_* = 27 μm, and *F* = 16 J/cm^2^) [141]. The experiments showed that the average rate decreased as the repetition rates increased. Specifically, the ablation rates were 0.69, 0.51, and 0.48 µm/pulse at 10, 100, and 500 Hz, respectively. The decline in the removal rate can largely be attributed to the fact that, at the same fluence, the laser-induced plasma expands in size and brightness as the repetition rate rises, intensifying the plasma-defocusing effects related to non-equilibrium ionization [151]. Non-equilibrium ionization causes the laser beam to diverge, reducing the intensity at the sample surface. Generally, increasing the repetition rate initially improves ablation efficiency and depth due to heat accumulation. However, exceeding an optimal threshold leads to excessive heat, surface damage, and reduced quality, making careful optimization essential for high-precision microfluidic structures, as discussed next.

**Figure 9:**
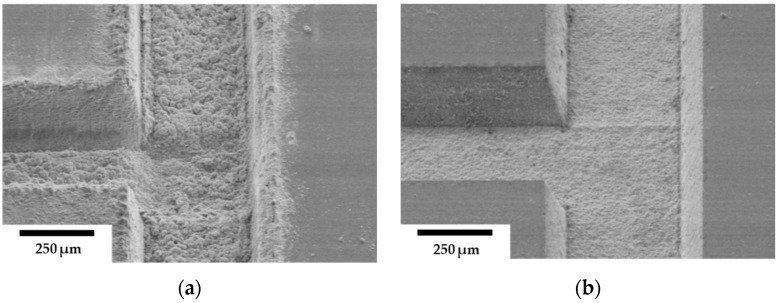
SEM images of T-shaped microfluidic channels produced in soda–lime glass using (**a**) a simple fs laser; (**b**) bi-burst mode. There is clear improvement in the ablation quality with the bi-burst regime compared to standard ablation [154]. Adapted with permission from reference [154].

The impact of heat accumulation at high repetition rates may not only affect the removal rate but also influence the etching quality, as reported in several studies. The effect of repetition rate (5 Hz–200 kHz) on soda–lime glass micromachining was investigated using a 1030 nm laser (*t_p_* = 500 fs, *d_L_* = 3.8 μm, and *F* = 112 J/cm^2^) [150]. The results indicated that all the holes exhibited excellent quality from 5 Hz to 20 kHz, with smooth inner surfaces and consistent hole diameters. However, at repetition rates of 25 and 50 kHz, a conical HAZ appeared on the glass surface. At repetition rates of 100 kHz and above, the glass deformed, and the holes collapsed due to a glass flow at approximately 50 μm below the surface. This morphological change is likely due to heat accumulation, as the time between consecutive pulses shortens, causing the glass to reach its softening temperature (around 700 °C for soda–lime glass), altering the hole’s geometry and potentially leading to collapse. In another study, the effect of the repetition rate on the removal quality of borosilicate glass was examined using a Ti:sapphire laser (*t_p_* = 35 fs, *λ* = 800 nm, *d_L_* = 27 μm, and *F* = 16 J/cm^2^) [141]. The results demonstrated a clear correlation between repetition rate and ablation quality, with the damage area increasing as the repetition rate rose from 10 to 500 Hz. At the base of the ablated area, the outer zone developed a ripple structure, with the period becoming larger toward the center. These laser-induced periodic surface structures may not arise from the interaction between the incident wave and surface-scattered waves [152] but rather from the relaxation of latent stresses in the glass surface during the melt layer’s re-solidification [153]. At high repetition rates (500 Hz), microcracks formed around the ablated craters, likely due to the interaction of the laser-induced plasma with successive pulses, affecting the surrounding material and the propagation of the plasma shockwave [58]. To enhance the quality of glass microfluidic parts, one study explored burst-mode femtosecond laser processing on soda–lime glass [154]. The burst mode was compared to standard ablation techniques using a Yb:KGW laser (*t_p_* = 200 fs, *λ* = 1030 nm, and *PRR* = 1–1000 Hz), demonstrating superior results in fabricating T-shaped microchannels with well-defined walls and corners (Figure 9a,b). This method significantly enhanced the surface roughness, reducing the root mean square (RMS) values from several micrometers to sub 500 nm.

#### 3.3.5. Effects of Laser Wavelength in Ultrashort-Pulse Micromachining

**Figure 10:**
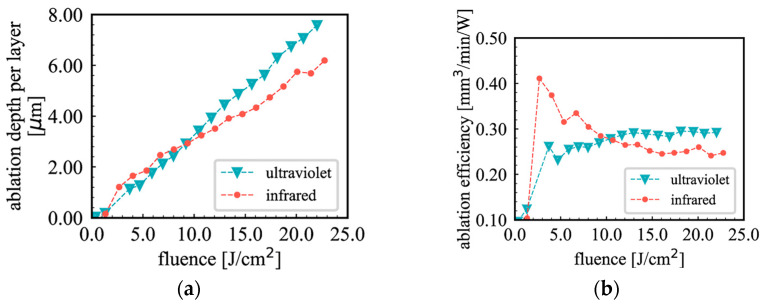

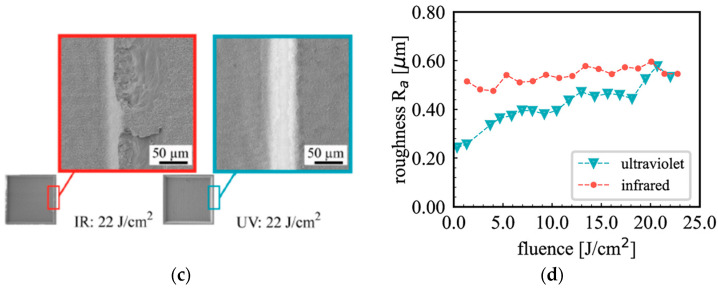
(**a**) The ablation depth per layer of fused silica using IR and UV lasers at the fluence range of 1– 23 J/cm^2^. (**b**) The ablation efficiency of IR and UV wavelengths vs. fluence. (**c**) The quality of the cavity edge using IR and UV lasers at a fluence of 22 J/cm^2^. (**d**) The effect of wavelengths and fluence on the roughness, Ra, of the ablated cavity [146]. Adapted with permission from reference [146].

For ultrashort lasers, nonlinear absorption mechanisms, like multiphoton absorption, become increasingly significant at specific wavelengths, affecting ablation efficiency. Shorter wavelengths can amplify these effects, allowing for more efficient material removal. Recently, Yang et al. explored the impacts of UV (343 nm) and IR (1030 nm) beams on etching depth and efficiency using an ultrashort-pulse laser (*t_p_* = 220 fs, *PRR* = 50 kHz, *d_L_* = 14.5 μm, and *V_scan_* = 100 mm/s) with fused silica as the substrate [146]. The IR laser achieved a notable removal depth per layer at low fluences, while the UV laser primarily enhanced depth at higher fluences, as shown in Figure 10a. The IR laser exhibited higher efficiency than the UV laser at low fluences, peaking at 0.41 mm^3^/min/W (for 3 J/cm^2^) before declining to 0.24 mm^3^/min/W, with further increases in fluence up to 23 J/cm^2^ (Figure 10b). Conversely, the performance of the UV laser increased slightly (0.26 mm^3^/min/W) with fluence up to 3 J/cm^2^ and then stabilized at higher fluences, reaching 0.30 mm^3^/min/W (Figure 10b). The enhanced ablation efficiency at shorter wavelengths with high fluence may result from a decrease in the effective penetration depth and the ablation threshold fluence as the wavelength decreases, given that photon energy is inversely proportional to wavelength. Thus, selecting the optimal wavelength is vital for maximizing process efficiency [155].

For precise microfabrication tasks, the laser wavelength is essential in determining the resolution and quality of the structures created. A study investigating the effect of wavelength on ablation quality using a 220 fs pulsed laser was conducted [146]. Two wavelengths, 1030 nm (infrared) and 343 nm (ultraviolet), were used for cavity ablation of fused silica. The results highlighted a substantial improvement in surface quality when employing the ultraviolet wavelength at a fluence of 22 J/cm^2^ compared to the infrared wavelength, as illustrated in Figure 10c. Chipping was observed at the ablated edge for the IR wavelength, whereas the UV-ablated cavity demonstrated a better edge quality. This can be attributed to the lower ablation threshold of the UV wavelength (less than 1 J/cm^2^) in comparison to that of the IR wavelength (3 J/cm^2^), along with the higher photon energy associated with the UV wavelength [97]. The UV beam, distinguished by its high photon energy, facilitates electron excitation across the bandgap of fused silica and minimizes heat transfer. The heat generated during etching is confined to the processing region, which increases thermal stress. Regarding surface roughness, the roughness (Ra) of the ablated cavity was lower for the UV wavelength than for the IR, as shown in Figure 10d. For example, at a fluence of 10 J/cm^2^, an Ra of 0.40 μm was achieved using the ultraviolet wavelength, while the Ra measured 0.55 μm with the infrared beam. Consequently, smoother microchannels can be fabricated using shorter wavelength beams in femtosecond-pulse laser processing.

#### 3.3.6. Effects of Glass Type in Ultrashort-Pulse Micromachining

The ablation efficiency of ultrashort lasers on different glass types is influenced by the glass’s chemical composition, thermal properties, and optical absorption. A study on the effects of soda–lime, sapphire, alkali-free alumina–borosilicate (AF32), and fused silica on removal depth using a Yb-doped laser (*t_p_ =* 500 fs, *λ* = 1030 nm, *PRR =* 10 kHz, *d_L_* = 3.8 μm, and *F* = 289 J/cm^2^) revealed that sapphire and soda–lime exhibited greater microchannel depth (250 and 240 µm, respectively) compared to AF32 and fused silica (180 and 115 µm, respectively) [150]. This can be attributed to the higher ablation threshold of AF32 and fused silica compared to soda–lime and sapphire. The same study also examined the effect of glass types on etching quality, finding that fused silica exhibited a superior removal quality compared to the soda–lime, sapphire, and AF32 glasses [150]. The soda–lime, sapphire, and AF32 glasses showed significant cracking, with cracks extending up to 60 µm, 22 µm, and 10 µm, respectively, around 17 µm diameter holes, and a cone-shaped HAZ was also observed. In contrast, the holes in the fused silica showed only minor cracks (a few µm) and a smaller HAZ, which can be attributed to its distinct thermal properties. Glasses like fused silica, with a lower thermal expansion coefficient (0.5 × 10^−6^ K^−1^) [39], generally experience less thermal damage compared to soda–lime, sapphire, and AF32 glasses, which have higher thermal expansion coefficients (~8 × 10^−6^ K^−1^, ~5.5 × 10^−6^ K^−1^, and ~3.2 × 10^−6^ K^−1^, respectively) [156,157,158].

#### 3.3.7. Summary of Reviewed Studies of Ultrashort-Pulse Laser Micromachining

The reviewed studies on ultrashort-pulse laser micromachining underscore its exceptional precision and reduced thermal impact, essential for fabricating high-quality glass microfluidic structures, as summarized in Table 5. Research demonstrates that increasing laser fluence significantly enhances ablation depth and efficiency; however, excessively high fluences can compromise quality by inducing surface roughness and microcracking. Scanning speed plays a critical role, with optimal speeds maximizing channel depth while maintaining quality; deviations lead to either thermal damage or insufficient ablation. Notably, shorter pulse durations, such as 35 fs, markedly improve both efficiency and surface smoothness by concentrating energy delivery and minimizing heat diffusion. The repetition rate must be carefully managed, as higher rates can boost material removal but risk introducing HAZs and defects beyond certain thresholds. Wavelength selection also influences outcomes, with ultraviolet lasers providing superior quality at higher fluences than infrared lasers, which are more efficient at lower fluences. Furthermore, the choice of glass type affects both the achievable depth and the quality of the micromachined features, with materials like fused silica offering excellent quality due to their low thermal expansion properties. These findings highlight the necessity for precise control and optimization of processing parameters in femtosecond laser micromachining to achieve the desired balance between efficiency and quality in glass microfluidic applications.

### 3.4. Comparison of Long, Short, and Ultrashort Laser Processing in Glass Micromachining

The laser pulse duration is a key parameter in the laser ablation process. Significant differences exist between long pulse durations (≥nanoseconds), short pulse durations (picoseconds), and ultrashort pulse durations (femtoseconds) concerning laser-based glass micromachining [50]. When a long laser pulse interacts with a target material, the surface is initially heated by the absorbed energy, leading to melting and vaporization. For long pulses, excited electrons transfer energy to the lattice throughout the duration of electron excitation, maintaining equilibrium between the electrons and the lattice. The laser pulse heats the material to its melting temperature during its duration [26]. Due to the longer linear absorption processes, the absorption length is significantly larger than for short pulses, resulting in deeper melt depths. The high temperatures generated can create a significant temperature gradient around the laser focal point, causing thermal stresses that may result in side effects, such as edge collapses, cracks, and scum during processing [106]. In contrast, short (picosecond) laser pulse interactions with the target material primarily involve energy absorption by free electrons via inverse Bremsstrahlung [50]. This leads to a transient nano-equilibrium state, where the electron temperature (Te) exceeds the lattice temperature (Ti). Although there is some energy exchange between the electrons and the lattice via heat conduction, the electrons are rapidly cooled, minimizing the mechanical and thermal damage to the ablated area. For ultrashort-pulse lasers, the pulse duration is much shorter than the time required for the energy transfer between the lattice and the free electrons. This results in the generation of very high temperatures and pressures at a shallow depth (on the order of microns), enabling a highly precise and smooth material removal process with a significantly smaller HAZ compared to that caused by longer pulses [50].

A study comparing the results obtained when soda–lime glass microchannels were fabricated with pulsed lasers in three pulse duration regimes (20 ns and 12 ps Nd:YVO_4_ lasers and a 100 fs Ti:sapphire laser) was reported [159]. To achieve microchannels with a 2:1 aspect ratio, nine scans with the nanosecond laser were applied at 50 mm/s to obtain a channel depth of 8.7 µm, compared to only one scan for the picosecond and femtosecond lasers at 20 mm/s. The surface roughness of the three channels was measured as 178.7 nm, 1028.3 nm, and 1016.3 nm for the nanosecond (20 ns)-, picosecond (12 ps)-, and femtosecond (100 fs)-pulse lasers, respectively. The surface roughness of the picosecond and femtosecond channels was higher than those manufactured with the nanosecond pulse. This is due to the explosive nature of ablation with short and ultrashort pulses, where the material is rapidly vaporized. The removal efficiencies for the three pulse regimes were compared in another study, with the results indicating that the femtosecond laser achieved the highest efficiency, ranging from 2.0 to 4.0 mm^3^/(W·min), with chip sizes of less than 5 µm [160].

**Figure 11:**
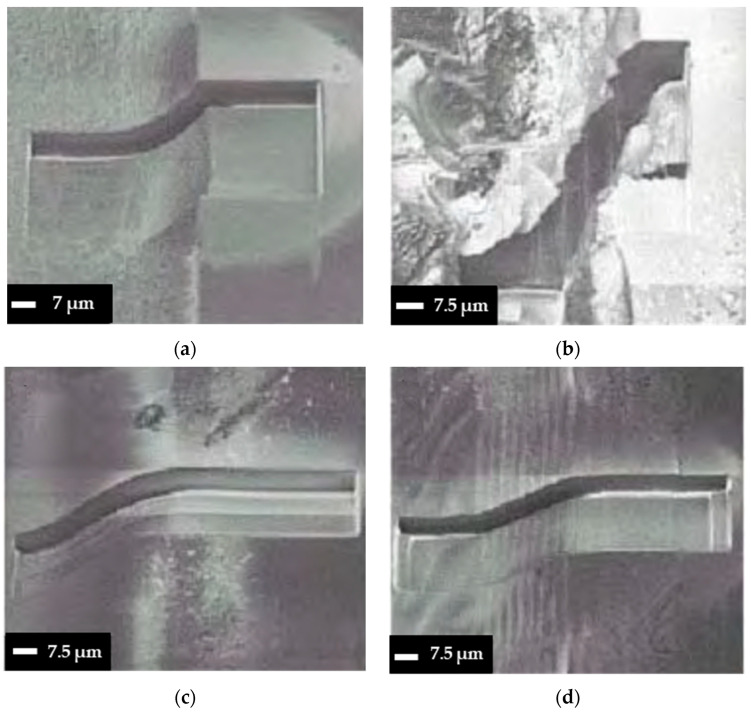
Cross-sectional micrographs of UV-grade, fused silica microchannels fabricated using (**a**) a 200 fs Ti:sapphire laser operating at 800 nm; (**b**) a 40 ns, 266 nm, diode-pumped, solid-state laser; (**c**) a 20 µs, 10.6 μm, CO_2_, pulsed laser; (**d**) a 150 ns, 9.25 μm, pulsed, Q-switched, CO_2_ laser [161]. Adapted with permission from reference [161].

Further investigation was carried out to compare the effects of long and ultrashort pulses on the formation of microchannels in UV-grade glass using 20 µs and 150 ns CO_2_ lasers, a 40 ns solid-state laser, and a 200 fs Ti:sapphire laser [161]. The femtosecond laser processing of fused silica showed minimal thermal damage around the microchannel, with a surface roughness of 1.25 µm (Figure 11a). In contrast, using the 40 ns laser resulted in an irregular, poorly defined wall geometry and a rough surface with a roughness of 19.97 µm (Figure 11b). For the CO_2_, microsecond-pulse laser process, significant thermal interaction, melting around the sidewalls, and debris formation around the removal area were observed, resulting in a surface roughness of 1.65 µm (Figure 11c). The 150 ns pulsed CO_2_ laser process produced a surface with diffraction imprinting and a roughness of 2.72 µm, possibly due to plasma formation, with no visible microcracks or subsurface damage (Figure 11d). The laser ablation threshold of the same material was investigated using three different pulse durations: 500 fs, 10 ps, and 20 ns, with wavelengths of 1030 nm for the 500 fs and 10 ps pulses and 1064 nm for the 20 ns pulse [97]. The experiment indicated reduced ablation threshold fluence as the laser pulse duration decreased, with values of 1054, 11.02, and 3.60 J/cm^2^ for 20 ns, 10 ps, and 500 fs, respectively. This reduction in threshold fluence leads to an enhancement in the quality of the fabricated structures. However, similar threshold fluences for barium alumoborosilicate glass were reported when using a 130 fs, 800 nm laser and a 10 ns, 266 nm laser, with values of 1.6 J/cm^2^ and 2.4 J/cm^2^, respectively [162]. This similarity may result from the effect of the 266 nm beam at nanosecond durations on the etching process, as discussed in Section 3.1.5.

However, Wang and colleagues reported that diamond microchannels fabricated with UV nanosecond lasers had a superior quality compared to those produced using IR picosecond laser pulses [79]. The channels created by the 12 ns laser were free from cracks and chipping, while significant cracks and chipping were observed at the ends of microgrooves made by the 10 ps laser. The team attributed these differences to the peak power densities, calculated to be 2.0 × 10^9^ W/cm^2^ for the nanosecond laser and 1.6 × 10^11^ W/cm^2^ for the picosecond laser. Consequently, the peak power density of the picosecond laser is approximately two orders of magnitude higher than that of the nanosecond laser. As a result, the temperature of the diamond material irradiated by the picosecond laser is significantly higher than that of the nanosecond laser. The thermal diffusion time is on the order of 10^−12^ s [91], which is much shorter than the pulse duration of the nanosecond laser. Thus, heat in the irradiated area from the nanosecond laser can diffuse to the unirradiated area, whereas heat from the picosecond laser cannot spread as easily. This difference leads to lower thermal stress in the diamond during nanosecond laser processing compared to picosecond laser processing. Regarding the removal rate, the results indicated that the nanosecond laser achieved a rate more than 10 times greater than that of the picosecond laser.

Femtosecond and picosecond laser pulse processes were compared using BK7 glass as the substrate [120]. A mode-locked, Yb-fiber laser, operating at 1035 nm, 250 kHz, with pulse durations ranging from 400 fs to 18 ps, was utilized to investigate the ablation rate. The results indicated that the highest rates were obtained at longer pulse durations in the 10–18 ps range, approximately 0.8 mm^3^/(W·min), compared to only 0.1 mm^3^/(W·min) with 400 fs pulses. The authors attributed this to the absence of free electrons at longer pulse durations, resulting in higher threshold fluences and, consequently, deeper penetration depths. Operating at sub-ps pulse durations causes breakdown effects due to nonlinear interactions with the glass. Concerning the ablation quality in the laser processing of BK7 glass under different pulse durations, two laser systems were employed to fabricate microchannels [161]. When using the 200 fs Ti:sapphire laser, a channel with a surface roughness of 0.97 μm was observed, alongside a significant vertical crack on the left side and surface sub-structuring on the right side. In contrast, when utilizing the 40 ns, 266 nm laser, the microchannel showed no cracks or subsurface damage, with a surface roughness of 0.93 μm.

Ablation processes for both femtosecond and picosecond pulse durations have also been explored in fused silica glass [110]. Using an Nd:YVO_4_ laser emitting 10 ps pulses at a 1064 nm wavelength and a Yb:YAG laser operating at 320 fs pulses at a 1030 nm wavelength, the findings indicate that the minimum removable glass layer thickness was sub-micrometer, measuring 0.94 μm for the femtosecond pulses. In contrast, the minimum removable thickness for the picosecond pulses was 12.95 μm. This increase is mainly due to the less localized energy absorption in the material with picosecond pulses, which exhibit a lower peak intensity and weaker nonlinear absorption than femtosecond pulses. Consequently, a higher process resolution and surface quality can be attained with femtosecond pulses. Surface roughness measurements revealed a value of 0.12 μm after optimizing the scanning speed (5.6 m/s) for the femtosecond pulses, while the roughness measured 0.59 μm at the optimal scanning speed of 1 m/s for the picosecond pulses.

The characteristics of long-, short-, and ultrashort-pulse lasers for glass micromachining have been studied. Table 6 summarizes the comparison of these pulse durations in the processing of glass material.

## 4. Discussion

This section discusses the impacts of laser parameters and highlights the various benefits of pulsed laser micromachining for glass-based microfluidics. Section 4.1 provides a comparative analysis of long-, short-, and ultrashort-pulse laser regimes in glass micromachining, detailing how key parameters such as fluence, scanning speed, pulse duration, repetition rate, wavelength, and glass type influence ablation efficiency and quality for microfluidic applications. Section 4.2 outlines the advantages of pulsed laser micromachining for glass microfluidics across various applications. Together, these subsections illustrate the significant impact of pulsed laser techniques on microfluidic technology.

### 4.1. Comparative Analysis of Pulsed Laser Regimes in Glass Micromachining

This section compares the three pulsed laser regimes—long, short, and ultrashort—assessing their performance across key parameters like fluence, scanning speed, pulse duration, repetition rate, wavelength, and glass type to identify optimal ablation conditions. Table 7 provides a comprehensive overview of the effects of key laser processing parameters across the three pulsed laser regimes (long, short, and ultrashort) in glass micromachining for microfluidic applications. The table synthesizes findings from multiple studies, highlighting optimal conditions for ablation efficiency and quality and notable trends. For instance, higher fluence enhances efficiency across all the regimes but compromises quality, causing thermal damage in long pulses and plasma shielding in short and ultrashort pulses. Scanning speed presents a trade-off, where moderate speeds maximize depth while excessive slowness risks thermal effects, particularly in long pulses. Shorter pulse durations in the picosecond and femtosecond regimes improve efficiency and quality by minimizing heat diffusion, unlike longer nanosecond pulses, which increase the depth at the cost of thermal damage. The repetition rate boosts efficiency through heat accumulation but degrades quality at high levels, especially in ultrashort pulses. The effects of wavelength vary, with shorter wavelengths favoring precision in long pulses and quality in ultrashort pulses, while glass type influences outcomes based on ablation thresholds and thermal properties. This comparative analysis emphasizes the necessity for parameter optimization tailored to specific microfluidic fabrication goals.

### 4.2. Pulsed Laser Micromachining Benefits for Glass Microfluidics

Pulsed laser micromachining revolutionizes glass microfluidic fabrication by utilizing long- to ultrashort-pulse lasers. This method surpasses traditional techniques, like photolithography and wet etching, offering enhanced precision, minimized thermal damage, quicker prototyping, cost efficiency, versatility, and the integration of complex functionalities [163,164]. These advancements boost device performance and application diversity. Therefore, glass-based microfluidic devices, fabricated by laser micromachining, leverage glass’s exponential properties to be used in a wide range of applications, which can be categorized as follows. First, biomedical applications include point-of-care diagnostics, single-cell analysis, tissue engineering, and drug discovery. Second, chemical and analytical applications, such as microreactors and micromixers, chromatography and separation processes, and optical detection systems. Third, environmental monitoring, such as real-time monitoring and sensors for detecting contaminants. Fourth, emerging and interdisciplinary applications, such as lab-on-chip systems and optofluidic uses, where integrated optical components enable advanced sensing and imaging in different fields. This section details each advantage with applications, showcasing recent studies’ contributions to microfluidic technology.

#### 4.2.1. High Precision and Resolution

Laser micromachining creates precise microstructures crucial for microfluidic devices. This precision arises from direct laser ablation, with Costa et al. demonstrating feature sizes as small as 3 μm for single-cell analysis using a femtosecond laser (*t_p_* = 160 fs, *λ* = 800 nm, *PRR* = 250 kHz, *V_scan_* = 0.1–0.5 mm/s, *P_avg_* = up to 1.4 W) [165]. Wlodarczyk et al. reported creating microchannels 15 to 58 μm deep in borosilicate and achieving a surface roughness of as low as 0.8 μm, ensuring smooth flow and reducing clogging risks, as shown in their picosecond-pulse laser research [14]. Achieving these satisfactory resolutions without masks cuts fabrication time and cost, boosting research efficiency. In addition, Fan et al. also fabricated microchannels 400 μm wide and 240 μm deep in soda–lime glass, highlighting the versatility of high-quality microfluidic mixing profiles [100]. This is critical for high-density arrays in drug discovery and genomics, where parallel processing enhances throughput [166].

**Figure 12:**
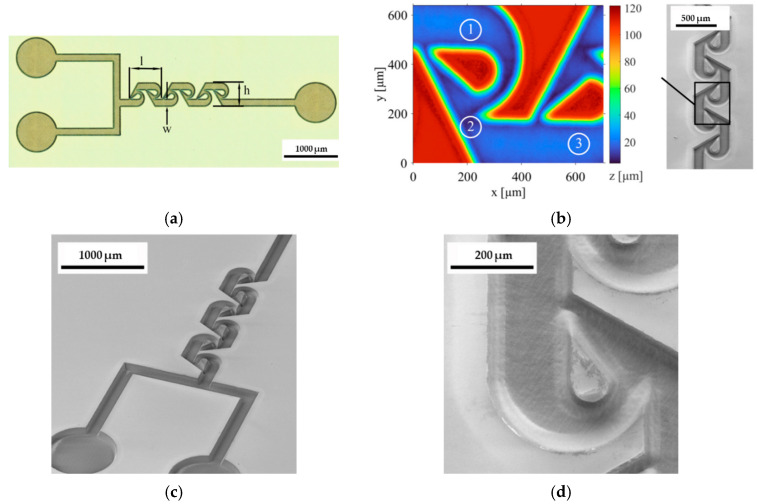
(**a**) The Tesla mixer, made using UV femtosecond laser ablation, measures 802.47 μm in length, 177.92 μm in width, and 597.95 μm in height. (**b**) The average surface roughness was assessed from three areas (1–3). (**c**,**d**) The SEM images of the mixer, captured at various magnification scales [146]. Adapted with permission from reference [146].

More recently, Yang et al. demonstrated the fabrication and performance evaluation of a fused-silica-based Tesla micromixer using ultraviolet femtosecond laser ablation (*t_p_* = 220 fs, and *λ* = 343 nm), which serves applications across a wide range of microfluidic platforms in biology, chemistry, tissue engineering, and lab-on-chip systems [146]. The mixer, characterized by bottleneck features as narrow as 260 µm, was produced using a fluence of 14 J/cm^2^, 65% pulse overlap, and a 75° hatch rotation over 37 ablation layers to ensure geometrical fidelity throughout the depth. As shown in Figure 12, scanning electron microscopy confirmed the high structural integrity of the device, with well-preserved curvature, sharp edge definition, and no evidence of chipping. Dimensional deviations remained below 1.2%, and the surface roughness was measured at 0.48 μm, consistent with earlier single-layer ablation results obtained under the same fluence.

#### 4.2.2. Minimal Thermal Damage

A key advantage of ultrashort-pulse lasers is minimal thermal damage, which limits the HAZ. Preserving glass integrity is essential for high-purity, durable applications, like biomedical devices. A study found that laser-welded devices withstand temperatures up to 620 °C, demonstrating robustness for sterilization and high-temperature operations [14]. Sugioka et al. highlighted that femtosecond lasers reduce thermal effects through nonlinear absorption, improving the quality of fabricated structures [31]. Ultrashort-pulse lasers minimize thermal damage, preserve glass integrity, and enable cold ablation due to nonlinear multiphoton absorption with negligible HAZs, vital for biomedical devices requiring high purity [31]. This preservation ensures that optical properties remain intact for detection systems, enhancing device reliability.

#### 4.2.3. Rapid Prototyping and Cost-Effectiveness

The direct-write capability of laser micromachining enables rapid prototyping, significantly shortening the development cycle. Unlike multi-step traditional methods, it allows for the quick generation of complex patterns, with a reported fabrication time of just 18 min for a complex pattern, highlighting its efficiency for low-volume production and research and development [14]. Also, Wlodarczyk et al. reported a fabrication time of just under two hours for a fully functional microfluidic device using a picosecond laser [115]. They used a picosecond laser (*t_p_* = 6 ps, *λ* = 515 nm, *PRR* = 40 kHz, *V_scan_* = 80 mm/s, and pulse energy = up to 62.8 μJ) [14,115]. In addition, García et al. highlighted the design flexibility and simplicity, which are crucial for rapid prototyping in medical applications [167].

For prototyping and small batch production, laser micromachining offers significant cost advantages over traditional methods. The elimination of masks, molds, and other tooling reduces upfront costs, and the flexibility of direct writing allows for easy design changes without additional expenses. Wlodarczyk et al. highlighted that the laser ablation method is suitable for rapid prototyping and low-volume manufacturing, implying reduced costs compared to conventional multi-step processes [14,115]. This benefits academic and small-scale research, accelerating innovation.

#### 4.2.4. Superior Cell Adhesion

Glass surfaces fabricated via laser micromachining exhibit superior cell adhesion compared to polymer substrates like PMMA, which is critical for biological applications. Konari et al. conducted experiments using a CO_2_ laser at speeds of 5–25 mm/s and a power of 4.5–18 W, comparing cell adhesion on laser-machined microchannels in glass, PDMS, and PMMA and finding that glass showed a higher cell density than PMMA after 30 min of incubation [21]. This property enhances the suitability of glass-based devices for tissue engineering and in vitro cell studies, where biocompatible surfaces enhance experimental outcomes.

#### 4.2.5. High-Aspect-Ratio Microchannels

Laser micromachining enables the creation of microchannels with high aspect ratios, which are challenging to achieve with conventional techniques. High-aspect-ratio channels are essential for applications requiring long, narrow passages, such as separation processes in chromatography or reactors needing an increased surface area [168]. Recent research by Tan et al. achieved an aspect ratio of approximately 30 for 3D microchannels in 10 mm thick silica glass using water-assisted femtosecond laser drilling with simultaneous spatiotemporal focusing, enhancing the capillary electrophoresis efficiency [169]. This capability supports complex 3D architectures, increasing device functionality.

#### 4.2.6. Integration of Sensors and 3D Structures

An unexpected yet significant benefit is the ability to integrate sensors and create complex three-dimensional structures within glass substrates [170]. This integration allows sensors to be embedded for real-time monitoring, expanding device functionality. Wlodarczyk et al. demonstrated embedding small 3D objects, including sensors, in their laser-fabricated devices, enabling in situ measurements during fluid flow experiments [14]. Sugioka et al. further noted the integration of micromechanical, microelectronic, and micro-optical components, broadening the scope for advanced applications [31]. This is vital for environmental monitoring and biomedical diagnostics, enabling point-of-care testing with rapid results.

**Figure 13:**
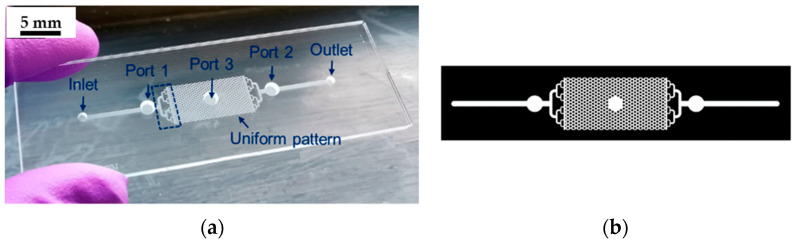
(**a**) A precision-engineered glass microfluidic device fabricated using advanced laser technology simultaneously integrates off-the-shelf fiber optic sensors to measure pH and pressure; (**b**) a diagram of the complete microfluidic pattern [171]. Adapted with permission from reference [171].

On the other hand, a recent study subsequently demonstrated a significant advancement in lab-on-chip technology by enabling the real-time, in situ monitoring of pH and pressure within laser-fabricated glass microfluidic devices; see Figure 13 [171]. Designed for embedded diagnostics, the system allows the dynamic tracking of fluidic and chemical processes under a controlled flow, making it highly suitable for biomedical and environmental sensing applications. The device was fabricated from borosilicate glass using a picosecond-pulse laser to ablate microfluidic channels and drill precise 2.8 mm ports for sensor integration. A second glass slide was then bonded to the device via laser micro-welding to seal it. Commercially available fiber optic pH and pressure sensors were embedded into these ports using custom-designed connectors fabricated with a stereolithography 3D printer [171].

#### 4.2.7. Reduced Use of Hazardous Chemicals

Laser micromachining minimizes the use of dangerous chemicals, as it does not require the hazardous substances often used in traditional glass fabrication methods, like photolithography and wet etching. Wlodarczyk et al. noted that the laser ablation method eliminates the need for projection masks, dangerous chemicals, and additional expensive tools [14]. Aralekallu et al. highlighted the environmental benefits, emphasizing the suitability for sustainable manufacturing practices [11].

#### 4.2.8. Hermetically Sealed Devices

Laser micromachining can produce hermetically sealed devices, ensuring contamination-free operations. Kim et al. measured the bonding strength of a glass microfluidic device fabricated by femtosecond laser micromachining and direct welding, finding it to be approximately 1.45 ± 0.15 MPa, sufficient for withstanding typical microfluidic operation pressures [172]. The femtosecond laser used 10 μJ for patterning, 2.7 μJ for welding, a 500 kHz repetition rate, and speeds of 100 mm/s and 20 mm/s. This capability simplifies manufacturing and enhances reliability in sensitive applications like drug delivery.

Table 8 highlights the benefits, applications, main features, and laser parameters, such as pulse duration (*t_p_*), wavelength (*λ*), repetition rate (*PRR*), scanning speed (*V_scan_*), and fluence (*F*), of laser-based glass micromachining.

## 5. Conclusions

This section concludes the review by summarizing key insights and future directions. Section 5.1 briefly explains how to optimize each of the process parameters in various pulse regimes to enhance glass micromachining efficiency and quality, making pulsed laser micromachining transformative for microfluidics. Section 5.2 proposes research directions, such as improving beam shaping, incorporating hybrid materials, and investigating 3D architectures, along with interdisciplinary applications (such as quantum photonics), to boost scalability and foster industrial adoption.

### 5.1. Summary

Pulsed lasers in three regimes—long-pulse (≥nanoseconds), short-pulse (picoseconds), and ultrashort-pulse (femtoseconds)—significantly impact the fabrication of microfluidic components, particularly microchannels in glass substrates. The efficiency and quality of material removal during laser ablation, influenced by factors such as pulse duration, fluence, scanning speed, repetition rate, wavelength, and glass types, are crucial for producing high-performance and precise glass-based microfluidic devices, as summarized below:

- Fluence is a key parameter because it directly affects the material removal rate, ablation depth, and precision. Across all three regimes, high fluence can accelerate material removal and create deeper cuts by delivering high energy, which increases local heat buildup, lowers the threshold fluence, and deepens optical penetration. However, if the fluence is too high, it may lead to undesirable effects, such as efficiency stabilization due to plasma shielding, thermal damage, cracks, and increased surface roughness. On the other hand, low fluence can improve removal precision but may also extend the fabrication time for microfluidic devices. These findings highlight the impact of high fluence on removal quality, making it crucial to optimize fluence in order to produce high-quality microfluidic channels on glass substrates with high efficiency.
- According to the research findings, scanning speed also significantly impacts the removal process across all three laser pulse regimes. When scanning speeds are too slow, more laser energy is absorbed, leading to excessive heat accumulation, which can cause thermal damage, such as cracking or surface degradation, negatively affecting ablation quality. Regarding ablation efficiency, slower scanning speeds result in limitations due to an increased plasma shielding effect, particularly with short- and ultrashort-pulses. On the other hand, a significant increase in scanning speed may lead to the formation of very shallow microchannels, as the increased distance between laser pulses results in insufficient energy being delivered to a single spot. Therefore, scanning velocity should be optimized to prevent heat accumulation caused by excessively slow speeds and shallow microchannels resulting from high scanning velocities.
- Optimizing pulse duration ensures higher efficiency, minimal thermal damage, and precise material removal. With long pulses, deeper microchannels can be achieved by extending the pulse duration, as this allows for deeper beam penetration and more heating time. In the case of picosecond and femtosecond pulses, shorter pulses result in higher ablation efficiency due to the higher peak power intensity (W/cm^2^) associated with these pulses and the lower ablation threshold. Regarding ablation quality, a reduction in quality was observed at longer pulse durations, as this allows mechanical relaxation processes in the glass, leading to a molten and rough surface.
- Optimizing the repetition rate of pulsed lasers is essential for improving efficiency, quality, and precision in glass micromachining, particularly in microfluidic channel fabrication. A high repetition rate leads to high ablation efficiency due to increased local heat accumulation, which reduces the threshold fluence. However, excessive heat accumulation can negatively impact precision and surface quality. At a point, the efficiency may drop due to plasma shielding, especially with short and ultrashort pulses. On the other hand, if the repetition rate is too low, the energy may not be effectively utilized, resulting in reduced ablation efficiency and a slower process.
- The wavelength of the laser light influences its interaction with the glass material. Since different wavelengths are absorbed by the glass to varying degrees, choosing the appropriate wavelength is essential for effective micromachining. With long pulses, shorter wavelengths are more effective for precise material removal, providing high efficiency and minimal thermal effects, such as warping, melting, and thermal stress. In contrast, longer wavelengths are less absorbed by glass and require higher energy to remove the desired material, which may result in lower-quality ablation. Short pulses have the highest removal rate with longer wavelengths, as they allow the laser beam to penetrate deeper into the material and provide a more uniform energy distribution within the glass. However, this comes at the cost of reduced precision and control over the removal process. In the case of ultrashort pulses, highly efficient ablation and higher quality were observed when shorter wavelengths were used at high fluence due to a decrease in the effective penetration depth and ablation threshold fluence. Therefore, selecting the optimal wavelength is crucial for maximizing process efficiency.
- The type of glass significantly influences ablation efficiency, with factors such as chemical composition, melting point, and optical properties playing critical roles. In all three pulsed regimes, the findings show the impact of the glass type on the removal effectiveness and precision. Some glass types, such as soda–lime and borosilicate glass, exhibit higher removal effectiveness due to lower ablation thresholds than fused silica and sapphire. However, materials with high ablation thresholds show a better removal quality because they can handle higher laser intensities without damage.

The comparative analysis of pulsed laser regimes in glass micromachining demonstrates that ultrashort-pulse lasers deliver high precision and minimal thermal damage, making them optimal for crafting intricate microfluidic structures in glass. For glass-based microfluidics, pulsed laser micromachining offers significant advantages, including rapid prototyping, cost-effectiveness, and the creation of high-aspect-ratio microchannels that enhance cell adhesion. This technology also enables the integration of sensors and 3D structures into glass substrates while minimizing hazardous chemical use and facilitating the production of hermetically sealed devices for improved safety and reliability. Overall, pulsed laser micromachining stands out as a transformative approach in microfluidic device fabrication, providing unmatched precision, efficiency, and versatility.

### 5.2. Directions for Future Research

This section outlines key future research directions for the pulsed laser micromachining of glass-based microfluidic devices. The main directions are summarized in Table 9, and they are as follows:

- Technological Advancements: A strong focus on advancing beam shaping and scanning strategies is essential to improve precision, efficiency, and the ability to create complex microstructures. In addition, beam shaping can enhance the control over energy distribution, reducing thermal effects and enabling high-aspect-ratio microchannels. Thus, developing new scanning strategies could address current limitations, such as shallow channels at high speeds or excessive heat at low speeds, improving the design complexity for 3D structures.
- Material and Integration: Integrating hybrid materials and combining glass with polymers or other substrates seem likely to be key areas. This approach could lead to multifunctional devices with enhanced properties, such as improved conductivity or flexibility, which is particularly relevant for applications requiring diverse material characteristics.
- Process Optimization: Optimizing laser parameters, such as fluence, scanning speed, and pulse duration, is critical to balancing fabrication efficiency and quality for different glass types. As noted in the review, this is crucial given the trade-offs identified, such as thermal damage at high fluence or reduced depth at fast scanning speeds. Such optimization could involve developing models or simulation tools for real-time parameter adjustment, enhancing process control.
- Three-Dimensional Architectures and Optical Integration: Developing three-dimensional architectures using femtosecond laser processing appears promising, leveraging its unmatched flexibility for designing intricate 3D microfluidic channels and embedding optical features. This could enable advanced applications, such as high-resolution detection systems, particularly in fields like biomedicine. The review emphasizes the potential for integrating micromechanical, microelectronic, and micro-optical components without stacking or bonding substrates.
- Scalability and Industrial Adoption: Focusing on cost-effective and high-throughput fabrication methods to transition from laboratory-scale research to industrial manufacturing will be a main area for development. Recent studies demonstrate rapid manufacturing techniques using pulsed lasers, which could reduce costs and time, facilitating broader adoption.
- Addressing technological challenges, such as reducing initial equipment costs, simplifying operations through user-friendly systems and fewer steps, and improving reliability, is crucial for broader adoption. For instance, short-pulse micromachining (using a picosecond laser) with optimized process parameters can fabricate glass-based microfluidics with an efficiency and quality similar to ultrashort-pulse micromachining (using a femtosecond laser). Thus, the optimization of the process parameters enables the avoidance of the high cost required for femtosecond lasers, such as Ti:sapphire-based systems.
- Exploring interdisciplinary applications, such as quantum photonics, biomedical devices, and environmental monitoring, could expand the technology’s impact. For instance, interdisciplinary applications include single-cell analysis, where precise microfluidic channels enable high-resolution cell studies, and quantum sensing, where glass-based devices could integrate with quantum technologies for advanced detection [11,173]. These fields highlight the potential of laser-fabricated microfluidics in biology and physics.

## Figures and Tables

**Table 1:** Comparison of fabrication methods of glass-based microfluidics.

Fabrication Method	Description	Pros	Cons	Examples of Glass Types Used
Photolithography [12]	Uses UV light and mask to pattern photoresist, followed by etching	High precision and resolution for microchannels	Requires cleanroom facilities and a multi-step process increases complexity and cost	Borosilicate glass (e.g., Borofloat 33), soda–lime glass, and Foturan glass
Wet chemical etching [5]	Uses etchants like HF to remove glass, often with photolithography	Cost-effective, produces well-defined geometries, and is often paired with photolithography	Involves hazardous chemicals and requires precise control of etching conditions	Fused silica, soda–lime glass, and Foturan glass
Soft lithography [13]	Creates molds (e.g., PDMS) for shaping or bonding with glass	Simple and low-cost, facilitates replication, and hybridizes with glass bonding	Limited by polymer mold stability and requires some cleanroom facilities	Borosilicate glass (Pyrex)
3D printing [39]	Rapid prototyping without cleanroom, custom designs possible	Rapid prototyping, enables complex, custom designs, and no cleanroom is needed	Limited material compatibility and lower resolution compared to other methods	Fused silica and ultra-thin glass
Laser ablation [20]	Uses lasers to remove or modify glass, maskless approach	High precision; no cleanroom or masks required; creates complex 3D structures	High initial equipment cost; requires expertise to optimize laser parameters	Fused silica, borosilicate glass (e.g., Borofloat 33), quartz, and Foturan glass

**Table 2:** Key factors influencing laser-based glass micromachining.

Factors	Description	Abbreviations
Fluence	Energy delivered per unit area	*F* (J/cm^2^)
Scanning speed	Laser travel speed during processing	*V_scan_* (mm/s)
Pulse duration	Time across a pulse at its full-width half maximum (FWHM)	*t_p_* (s)
Repetition rate	Number of pulses per second	*PRR* (Hz)
Wavelength	Laser output wavelength	*λ* (nm)
Laser beam diameter	The diameter of the laser beam on the sample	*d_L_* (µm)
Peak power	Maximum peak power attained by a single pulse	*P_p_* (W)
Average power	Total average output power utilized in material processing	*P_avg_* (W)

**Table 3:** Summary of reviewed studies on key parameters influencing ablation efficiency and quality in long-pulse laser micromachining of glass.

Studied Effect	Laser Type	*t_p_*	*PRR* (kHz)	*V_scan_* (mm/s)	Glass Type	Notes
Fluence (*F*)	CO_2_ laser (*λ* = 10.6 μm, *d_L_* = 240 μm) [78]	300 ns	20	20	Quartz	Efficiency increased from 0.7 × 10^−3^ to 18 × 10^−3^ mm^3^/J as the *F* increased from 1.12 to 7.18 J/cm^2^.
	Nd:YAG laser (*λ* = 355 nm) [79]	12 ns	30	5	Diamond	Removal rate increased from 0.45 × 10^−10^ to 3.34 × 10^−10^ g/pulse as the *F* increased from 11.41 to 47.91 J/cm^2^.
	CO_2_ laser (*λ* = 10.6 μm, *d_L_* = 300 μm) [80]	45 μs	1	20	Fused silica	Ablation depth increased from 0.04 to 10 μm as the *F* increased from 3 to 5.8 J/cm^2^.
	Nd:YAG laser (*λ* = 355 nm, *d_L_* = 15 μm) [81]	30 ns	5	20	Soda–lime	Depth increased from 12 to 50 μm as *F* increased from 20 to 100 J/cm^2^, then saturated at 50 μm for fluences above 100 J/cm^2^.
	CO_2_ laser (λ = 10.6 μm, *d_L_* = 240 μm) [78]	300 ns	150	750	Quartz	Surface roughness (Sa) increased to 0.75 μm as *F* increased from 0.80 to 2 J/cm^2^.
	CO_2_ laser (*λ* = 10.6 μm, *d_L_* = 347 μm) [83]	N/A	5	2000	Soda–lime	Clear surface melting and solidification cracks form around the channels when the energy deposition rate exceeds 6.0 J/(cm^2^·s).
	Nd:YAG laser (*λ* = 266 nm, *d_L_* = 6 μm) [84]	10 ns	4.5	0.2–0.6	Borofloat	Crack-free channels at low energy (78 μJ).
Scanning speed (*V_scan_*)	Nd:YAG laser (*λ* = 266 nm) [86]	ns	5	5–10	Pyrex	Removal rate increased from 10 to 18 μm/pulse as *V_scan_* risen from 5 to 10 mm/s.
	CO_2_ laser (*λ* = 10.6 μm, *d_L_*= 400 μm) [22]	45 μs	20	100–600	K-PSFn214	Depth increased from 4 μm to 10 μm as *V_scan_* decreased from 600 to 100 mm/s.
	CO_2_ laser (*λ* = 10.6 μm) [21]	N/A	N/A	5–25	Microscope glass	Depth decreased from 55.8 μm to 22.4 μm as *V_scan_* increased from 5 to 25 mm/s.
	CO_2_ laser (*λ* = 10.6 μm, *d_L_* = 400 μm) [22]	50 μs	20	100–1200	K-PSFn214	Visible microcracks in channels at all applied *V_scan_*.
	CO_2_ laser (*λ* = 10.6 μm, *d_L_* = 62 μm) [89]	N/A	8	50–65	Fused silica	Surface roughness increased from 350 nm to 1500 nm with decreasing *V_scan_*.
pulse duration (*t_p_*)	CO_2_ laser (*λ* = 10.6 μm, *d_L_* = 300 μm) [80]	54–185 μs	1	N/A	Fused silica	Depth increased from 3.5 nm to over 8.2 μm as *t_p_* increased from 54 to 185 μs.
	CO_2_ laser (*λ* = 10.6 μm, *d_L_* = 266 μm) [91]	300–1000 μs	130	N/A	Fused silica	Depth increased from 5 μm to 44 μm as *t_p_* increased from 300 to 1000 μs.
	CO_2_ laser (*λ* = 10.6 μm, *d_L_* = 35 μm) [93]	1–200 μs	0.2	N/A	Fused silica, borosilicate	Shorter *t_p_* reduced HAZ and cracking.
	CO_2_ laser (*λ* = 10.6 μm, *d_L_* = 90 μm) [95]	5–90 μs	0.5	75	Fused silica	HAZ thickness increased from 3 μm to 9.6 μm with longer *t_p_*.
Repetition rate (*PRR*)	CO_2_ laser (*λ* = 10.6 μm, *d_L_* = 240 μm) [78]	300 ns	20–150	20	Quartz	Efficiency increased from 5 × 10^−3^ to 12 × 10^−3^ mm^3^/J as *PRR* increased from 20 to 150 kHz.
	CO_2_ laser (*λ* = 10.6 μm, *d_L_* = 300 μm) [80]	100 μs	0.2–3.2	20	Fused silica	A 500% depth increase with *PRR* from 300 Hz to 3200 Hz.
	Nd:YVO_4_ laser (*λ* = 1064 nm, *d_L_* = 20 μm) [23]	20 ns	8–16	200	Soda–lime	Bottom roughness increased from 12 μm to 19 μm as *PRR* increased from 8 to 16 kHz, with cracks and surface irregularities observed for *PRR* above 10 kHz.
Wavelength (*λ*)	Nd:YAG laser (*λ* = 355 and 266 nm) [86]	ns	N/A	10	Pyrex and sapphire	Removal rate at 266 nm: 23 μm/pulse (Pyrex), 0.8 μm/pulse (sapphire). At 355 nm, lower rates: 4 μm/pulse (Pyrex) and 0.4 μm/pulse (sapphire), with the 266 nm laser providing better precision.
Glass types	Nd:YVO_4_ laser (*λ* = 1064 nm, *d_L_* = 19 μm) [97]	20 ns	N/A	N/A	Soda–lime, borosilicate, fused silica, sapphire	Threshold values: 116 J/cm^2^ (soda–lime), 954 J/cm^2^ (borosilicate), 1054 J/cm^2^ (fused silica), 1100 J/cm^2^ (sapphire).
	CO_2_ laser (*λ* = 10.6 μm, *d_L_* = 35 μm) [93]	20 μs	0.2	N/A	Fused silica, borosilicate	Higher removal rate for borosilicate (up to 3.5 μm/pulse) compared to fused silica (2 μm/pulse).
	CO_2_ laser (*λ* = 10.6 μm, *d_L_* = 35 μm) [98]	N/A	1	N/A	Quartz, B270, Borofloat, Pyrex, soda–lime	Quartz microchannels exhibited smoother surfaces and fewer microcracks than other glass types.
	Nd:YVO_4_ laser (*λ* = 355 nm, *d_L_* = 1 μm) [65]	1.3 ns	5	100	Fused silica, diamond, sapphire	Cracking and deformation in fused silica glass, minor cracking, and minimal distortion in other glass types.

N/A means not available.

**Table 4:** Summary of reviewed studies on key parameters influencing ablation efficiency and quality in short-pulse laser micromachining of glass.

Studied Effect	Laser Type	*t_p_*	*PRR* (kHz)	*V_scan_* (mm/s)	Glass Type	Notes
Fluence (*F*)	Picosecond laser (*λ* = 355 nm, *d_L_* = 13.8 μm) [100]	10 ps	800	N/A	Soda–lime	Crater depth increased from 0.035 to 0.143 μm as *F* increased from 3.28 to 6.49 J/cm^2^
	Picosecond laser (*λ* = 355 nm, *d_L_* = 15 μm) [111]	10 ps	800	200	Borosilicate	Efficiency increased from 0.1 to 3.2 μm^3^/μJ as *F* increased from 2 to 3 J/cm^2^, then stabilized around 3.2 μm^3^/μJ for *F* values exceeding 3 J/cm^2^
	Picosecond laser (*λ* = 1064 nm, *d_L_* = 27 μm) [112]	13 ps	100	2	Borosilicate	Efficiency rose from 0.9 to 1.8 μm^3^/μJ as *F* increased from 5.5 to 12 J/cm^2^, then stabilized at approximately 1.6 μm^3^/μJ for *F* above 12 J/cm^2^
	Thin-disk laser (*λ* = 355 nm, *d_L_* = 10 μm) [114]	6 ps	20	100	Thin glass	Effective cutting speed increased from 5 to 19 mm/s as *F* increased from 10 to 15 J/cm^2^, then stabilized beyond 15 J/cm^2^
	Picosecond laser (*λ* = 515 nm, *d_L_* = 21 μm) [115]	6 ps	100	150	Borosilicate	Slight increase in average surface roughness from 1.55 µm to 1.72 µm as *F* increased from 16 to 33 J/cm^2^
	Picosecond laser (*λ* = 515 nm, *d_L_* = 24 μm) [14]	6 ps	20	40	Borofloat 33	Surface roughness remained around 1.6 µm, with minimal variation across *F* ranging from 11 to 31 J/cm^2^
	Thin-disk laser (*λ* = 343 nm, *d_L_* = 10 μm) [114]	6 ps	20	100	AF32 Eco thin glass	A small HAZ (<20 μm) appeared around holes produced at *F* values ranging from 20 to 37 J/cm^2^, with no microcracks observed
	Picosecond laser (*λ* = 1064 nm, *d_L_*= 60 μm) [79]	10 ps	200	5	Diamond	Cracks and chipping at *F* of 1.41 and 2.83 J/cm^2^
Scanning speed (*V_scan_*)	Picosecond laser (*λ* = 355 nm, *d_L_* = 15 μm) [111]	10 ps	653	200–1000	Borosilicate	Optimal *V_scan_* was 800 mm/s, achieving 5.75 μm^3^/μJ ablation efficiency, compared to 3.25 and 4 μm^3^/μJ at 200 and 1000 mm/s, respectively
	Picosecond laser (*λ* = 1064 nm, *d_L_* = 25 μm) [117]	<12 ps	50	5–360	Glass	Cracks and debris below 75 mm/s, cracks disappeared and debris remained between 75–150 mm/s, no cracks or debris above 150 mm/s
Pulse duration (*t_p_*)	Yb-fiber laser (*λ* = 1035, 517, and 345 nm, *d_L_* = 20 μm) [120]	1.5–14 ps	250	N/A	BK7	At *F* = 3.2 J/cm^2^ and 1035 nm, there is a 37% increase in etching rate with 1.5 ps pulses compared to 10 ps. At *F* = 4 J/cm^2^ and 517 nm, ablation rate of 0.65 mm^3^/(W·min) at 1.5 ps, 0.48 mm^3^/(W·min) at 14 ps. At *F* = 1.8 J/cm^2^ and 345 nm, ablation rate of 0.50 mm^3^/(W·min) at 1.5 ps, 0.10 mm^3^/(W·min) at 10 ps
	Picosecond laser (*λ* = 1053 nm, *d_L_* = 50 μm) [124]	0.4–60 ps	10	N/A	Fused silica	Smooth craters at 1.8 ps pulses, damage morphologies with local defects or vulnerable areas at longer *t_p_*
Repetition rate (*PRR*)	Picosecond laser (*λ* = 1000 nm, *d_L_* = 25 μm) [125]	<10 ps	500–2000	N/A	Glass	Removal rate reached 14 mm^3^/min at 2000 kHz, compared to 2 mm^3^/min at 500 kHz
	Nd:YVO_4_ laser (*λ* = 532 nm, *d_L_* = 3.4 μm) [126]	12 ps	200–1000	0.1	Fused silica	Depths of 1 mm, 500 µm, and 200 µm at 200, 400, and 1000 kHz, respectively, with a large HAZ (>100 µm at 400 kHz and 200 µm at 1000 kHz) around the entrance, and no HAZ at 200 kHz
	Picosecond laser (*λ* = 1064 nm, *d_L_* = 26.5 μm) [66]	10 ps	400–800	5000	Soda–lime	70% reduction in cutting efficiency at 800 kHz compared to 400 kHz
	Picosecond laser (*λ* = 1064 nm, *d_L_* = 27 μm) [110]	13 ps	400–650	1000	Borosilicate	Heat accumulation effects became more pronounced as the *PRR* increased from 400 to 650 kHz
	Picosecond laser (*λ* = 355 nm, *d_L_* = 13.6 μm) [100]	10 ps	500– 800	4000	Soda–lime	Irregular edges at higher *PRR* (600–800 kHz) compared to 500 kHz
Wavelength (*λ*)	Thin-disk laser (*λ* = 1030, 515, and 343 nm, *d_L_* = 22.5 μm) [114]	6 ps	400	2000	AF32 glass	Highest cutting speed (110 mm/s) was attained at 1030 nm, compared to 70 mm/s at 515 nm and 30 mm/s at 343 nm, with the best cutting quality observed at 343 nm
	Yb-Fiber laser (*λ* = 1035, 517, and 345 nm, *d_L_* = 25 μm) [120]	5 ps	250 and 500	N/A	BK7	The highest ablation rate, 0.25 mm^3^/(W·min), occurred at 1035 nm, compared to 0.21 mm^3^/(W·min) at 517 nm and 0.15 mm^3^/(W·min) at 343 nm
Glass types	Thin-disk laser (*λ* = 1030 nm, *d_L_* = 19 μm) [97]	10 ps	N/A	N/A	Soda–lime, fused silica, sapphire, borosilicate	Soda–lime and borosilicate glasses exhibited lower ablation thresholds of 9.54 and 9.40 J/cm^2^, respectively, compared to fused silica (11.02 J/cm^2^) and sapphire (13.05 J/cm^2^)

N/A means not available.

**Table 5:** Summary of reviewed studies on key parameters influencing ablation efficiency and quality in ultrashort-pulse laser micromachining of glass.

Studied Effect	Laser Type	*t_p_*	*PRR* (kHz)	*V_scan_* (mm/s)	Glass Type	Notes
Fluence (*F*)	Ti:sapphire laser (*λ* = 800 nm, *d_L_* = 27 μm) [141]	35 fs	0.01	N/A	Borosilicate	The ablation rate increased from 0.19 to 0.63 μm/pulse as the *F* rose from 0.9 to 3.5 J/cm^2^, stabilizing at 0.65 μm/pulse for *F* between 3.5 and 11 J/cm^2^
	Yb:KGW laser (*λ* = 1030 nm, *d_L_* = 20 μm) [142]	280 fs	60	100	Soda–lime	Groove depth increased from 55 to 90 µm as *F* rose from 9.5 to 23.5 J/cm^2^
	Femtosecond laser (*λ* = 1030 nm, *d_L_* = 7.9 μm) [144]	190 fs	1	100	Fused silica	Channel depth > 850 µm at 1958 J/cm^2^, compared to 150 µm at 244 J/cm^2^. Smooth sidewalls at 1101.7 J/cm^2^ and lower *F*, rougher sidewalls at higher *F* like 1958 J/cm^2^
	Femtosecond laser (*λ* = 343 nm, *d_L_* = 14.5 μm) [146]	220 fs	50	100	Fused silica	Topological changes or chipping at cavity edges as *F* increased from 5 to 22 J/cm^2^. Surface roughness: 0.37 µm at 5 J/cm^2^, 0.54 µm at 22 J/cm^2^
Scanning speed (*V_scan_*)	Yb:KGW laser (*λ* = 1030 nm, *d_L_* = 20 μm) [142]	280 fs	60	50–300	Soda–lime	Groove depth decreased to 40 µm as *V_scan_* increased to 300 mm/s, compared to 135 µm at 50 mm/s
	Yb:KGW laser (*λ* = 1030 nm, *d_L_* = 7.9 μm) [144]	190 fs	1	0.020–0.5	Fused silica	The channel depth increased from 400 to 750 µm as *V_scan_* rose from 20 to 150 μm/s, then decreased to 250 µm as *V_scan_* further increased to 500 μm/s
	Ti:sapphire laser (*λ* = 800 nm) [24]	100 fs	1	0.5–2	Quartz	At lower *V_scan_* (<1.5 mm/s), ablation quality improved, but at *V_scan_* below 0.5 mm/s, heat accumulation caused tiny cracks. At higher *V_scan_* (>1.5 mm/s), etching quality deteriorated
Pulse duration (*t_p_*)	Ti:sapphire laser (*λ* = 800 nm, *d_L_* = 27 μm) [141]	35–500 fs	0.01	N/A	Borosilicate	Ablation rate: 0.22 μm/pulse with 500 fs pulses and rough surface, 0.65 μm/pulse with 35 fs pulses and smooth crater
Repetition rate (*PRR*)	Yb-doped laser (*λ* = 1030 nm, *d_L_* = 3.8 μm) [150]	500 fs	0.005–200	N/A	Soda–lime	Etching depth increased from 147 to 350 µm as *PRR* rose from 5 Hz to 20 kHz with excellent quality, but beyond 20 kHz, the depth decreased, and a HAZ appeared on the surface
	Ti:sapphire laser (*λ* = 800 nm, *d_L_* = 27 μm) [141]	35 f	0.01–0.5	N/A	Borosilicate	Ablation rates were 0.69 µm/pulse at 10 Hz, 0.51 µm/pulse at 100 Hz, and 0.48 µm/pulse at 500 Hz, with microcracks forming at 500 Hz
Wavelength (*λ*)	Femtosecond laser (*λ* = 1030 and 343 nm, *d_L_* = 14.5 μm) [146]	220 fs	50	100	Fused silica	At low *F*, the IR laser achieved a significant removal depth per layer, while the UV laser enhanced depth at higher *F* and notably improved surface quality
Glass types	Yb-doped laser (*λ* = 1030 nm, *d_L_* = 3.8 μm) [150]	500 fs	10	N/A	Soda–lime, sapphire, borosilicate (AF32), fused silica	Sapphire and soda–lime exhibited higher microchannel depths (250 and 240 µm, respectively) than AF32 and fused silica (180 and 115 µm, respectively), although fused silica showed superior removal quality

N/A means not available.

**Table 6:** Comparison of long-, short-, and ultrashort-pulse laser processing of glass materials.

Feature	Material	Long-Pulse Laser (≥ns)	Short-Pulse Laser (ps)	Ultrashort-Pulse Laser (fs)
Ablation threshold	Soda–lime	High	Medium	Low [97]
	Borosilicate			
	Fused silica			
	Sapphire			
	Bariumalumo–borosilicate glass	Low	Low	Low [162]
Ablation efficiency	Soda–lime	Low	High	High [159]
	Sapphire	Low	Medium	High [160]
	BK-7	-----	High	Low [120]
	Diamond	High	Low [79]	-----
Ablation quality	Fused silica	Low	-----	High [161]
	BK-7	High	-----	Low [161]
	Diamond	High	Low [79]	-----
	Fused silica	-----	Low	High [110]
Thermal damage	Fused silica	High	-----	Low [161]
	BK-7			
Surface roughness	Soda–lime	Low	High	High [127]
	Fused silica	High	-----	Low [161]
	BK-7	Low	-----	Low [161]
	Fused silica		Low	High [110]

**Table 7:** Effects of parameters on glass micromachining across laser pulse duration regimes.

Parameter	Pulse Duration	Range Studied	Optimal for Efficiency	Optimal for Quality	Notable Trends
Fluence (*F*)	Long (≥ns) [78,79,80,81]	0.8–132.3 J/cm^2^	High *F* (e.g., 100 J/cm^2^)	Low *F* (e.g., 0.8 J/cm^2^)	Efficiency increases with *F*, while quality decreases due to thermal damage
	Short (ps) [100,111,112,114]	2–37 J/cm^2^	Moderate *F* (e.g., 3–15 J/cm^2^)	Low to moderate F (e.g., 3–20 J/cm^2^)	Efficiency increases to a threshold, then stabilizes due to plasma shielding, while quality degrades at high *F*
	Ultrashort (fs) [141,142,144,146]	0.9–1958 J/cm^2^	High *F* (e.g., up to 1958 J/cm^2^)	Low to moderate F (e.g., 5–1101 J/cm^2^)	Efficiency increases with *F*, but high *F* increases roughness and microcracking
Scanning speed (*V_scan_*)	Long (≥ns) [21,22,86,89]	5–1200 mm/s	Moderate (e.g., 10 mm/s)	High (e.g., 600 mm/s)	Slower speeds increase depth but risk thermal damage, while faster speeds improve quality
	Short (ps) [111,117]	5–1000 mm/s	Moderate (e.g., 800 mm/s)	High (e.g., >150 mm/s)	Efficiency peaks at optimal speed; slower speeds cause cracks, while faster speeds reduce depth
	Ultrashort (fs) [24,142,144]	20 μm/s–300 mm/s	Optimal (e.g., 150 μm/s)	Moderate (e.g., 1.5 mm/s)	Slower speeds maximize depth to a threshold, then stabilize, but risk heat buildup; faster speeds enhance quality
Pulse duration (*t_p_*)	Long (≥ns) [80,91,93,95]	5–1000 μs	Longer *t_p_* (e.g., 1000 μs)	Shorter *t_p_* (e.g., 5 μs)	Longer *t_p_* increases depth and expands HAZ, while shorter *t_p_* reduces thermal effects
	Short (ps) [120,124]	1.5–60 ps	Shorter *t_p_* (e.g., 1.5 ps)	Shorter *t_p_* (e.g., 1.5 ps)	Shorter *t_p_* enhances efficiency and quality by minimizing thermal diffusion
	Ultrashort (fs) [141]	35–500 fs	Shorter *t_p_* (e.g., 35 fs)	Shorter *t_p_* (e.g., 35 fs)	Shorter *t_p_* enhances efficiency and quality by reducing HAZ and enabling precise ablation
Repetition rate (*PRR*)	Long (≥ns) [23,78,80]	0.2–150 kHz	High (e.g., 150 kHz)	Low (e.g., 300 Hz)	Higher *PRR* boosts efficiency through heat accumulation but risks thermal damage, impacting quality
	Short (ps) [66,100,110,125,126]	200–2000 kHz	Moderate (e.g., 2000 kHz)	Low to moderate (e.g., <600 kHz)	High *PRR* increases efficiency but causes thermal damage above threshold
	Ultrashort (fs) [141,150]	5 Hz–200 kHz	Moderate (e.g., 20 kHz)	Low (e.g., <20 kHz)	Efficiency peaks at moderate *PRR*; high *PRR* increases HAZ and degrades quality
Wavelength (*λ*)	Long (≥ns) [86,96]	193–355 nm	Shorter *λ* (e.g., 266 nm)	Shorter *λ* (e.g., 193 nm)	Shorter *λ* improves absorption and precision, while longer *λ* increases thermal effects
	Short (ps) [114,120]	343–1035 nm	Longer *λ* (e.g., 1035 nm)	Shorter *λ* (e.g., 343 nm)	Longer *λ* enhances efficiency through deeper penetration; shorter *λ* improves quality
	Ultrashort (fs) [146]	343–1030 nm	Varies (IR low F, UV high F)	Shorter *λ* (e.g., 343 nm)	Shorter *λ* reduces roughness and improves quality; efficiency varies with *F*
Glass type	Long (≥ns) [65,93,97,98]	Soda–lime, borosilicate, fused silica, sapphire, quartz, B270, Pyrex, and diamond	Low threshold (e.g., soda–lime)	High threshold (e.g., quartz)	Low-threshold glasses are effective at ablation but may compromise quality, whereas high-threshold glasses improve quality
	Short (ps) [97]	Soda–lime glass, fused silica, sapphire, and borosilicate	Low threshold (e.g., soda–lime)	High threshold (e.g., fused silica)	Lower thresholds increase efficiency, while higher thresholds enhance quality
	Ultrashort (fs) [150]	Soda–lime, sapphire, borosilicate (AF32), and fused silica	Low threshold (e.g., soda–lime)	Low thermal expansion (e.g., fused silica)	Low threshold boosts efficiency; low thermal expansion reduces cracking and improves quality

**Table 8:** Detailed benefits of laser micromachining for glass-based microfluidics.

Benefit	Application	Highlighted Features	Laser Parameters (*t_p,_ λ, PRR, F, V_scan_*)
High precision and resolution	Fine, intricate designs (e.g., single-cell analysis, high-density arrays)	Feature sizes down to 3 μm, roughness ~0.8 μm	160 fs, 800 nm, 250 kHz, 0.1–0.5 mm/s, and up to 1.4 W [165]
Minimal thermal damage	Biomedical devices	Negligible HAZ, withstands up to 620 °C	Femtosecond, ultrashort-pulse, high peak intensity, not specified [31]
Rapid prototyping and cost-effectiveness	Prototyping, small batch production for research and development	Fabrication in <2 h, 18 min for pattern	6 ps, 515 nm, up to 40 kHz, up to 80 mm/s, and up to 62.8 μJ [14,115]
Superior cell adhesion	Better cell attachment on glass surfaces in tissue engineering and cell studies	Higher cell density vs. PMMA at 30 min	CO_2_ laser, 5–25 mm/s, 4.5–18 W, not specified [21]
High aspect ratio microchannels	Fabricates long, narrow channels with high depth-to-width ratio for separation processes and reactors	Aspect ratio of ~30 for 10 mm thick glass	40 fs, 800 nm, 1 kHz, and 4 mJ (spatiotemporal focusing, not specified) [169]
Integration of sensors and 3D structures	Real-time monitoring in various applications	Embedding sensors for in situ measurements	6 ps, 515 nm, up to 40 kHz, up to 80 mm/s, and up to 62.8 μJ [14]
Reduced use of hazardous chemicals	Sustainable manufacturing	No use of projection masks, dangerous chemicals	6 ps, 515 nm, up to 40 kHz, up to 80 mm/s, and up to 62.8 μJ [14]
Hermetically sealed devices	Contamination-free operations	Bonding strength: 1.45 ± 0.15 MPa	300 fs to 10 ps, 1300 nm, and 10 μJ for patterning, 2.7 μJ for welding, 500 kHz, 100 mm/s for patterning, and 20 mm/s for welding [172]

**Table 9:** Summary of future research directions.

Area	Focus	Key Challenge	Expected Outcome
Beam Shaping and Scanning	Enhance precision with advanced techniques	Controlling energy distribution	Complex, high-aspect-ratio structures
Hybrid Material Integration	Combining glass with polymers	Material compatibility	Multifunctional devices
Parameter Optimization	Fine-tuning fluence, speed, etc.	Balancing efficiency and quality	High-quality microchannels
3D Architectures	Optimize for intricate designs and optics	Integration complexity	Advanced detection systems
Industrial Scalability	Cost-effective and high-throughput methods	Scaling costs and processes	Industrial adoption
Technological Challenges	Reduce costs and improve accessibility	High equipment cost, expertise	Broader adoption
Interdisciplinary Applications	Expand into quantum and biomedical fields	Application-specific optimization	Broader impacts in emerging fields

## Data Availability

The original contributions presented in this study are included in the article. Further inquiries can be directed to the corresponding authors.
